# Hydrogen Bonds Induce Double-Well Spectroscopic Signatures in α‑Glycine

**DOI:** 10.1021/jacs.5c13223

**Published:** 2025-10-31

**Authors:** Noam Pinsk, Nimrod Benshalom, Michal Hartstein, Yael Diskin-Posner, Matan Menahem, Olle Hellman, Leeor Kronik, Omer Yaffe

**Affiliations:** † Department of Chemical and Biological Physics, 34976 Weizmann Institute of Science, Rehovot 76100, Israel; ‡ Department of Molecular Chemistry and Materials Science, Weizmann Institute of Science, Rehovot 76100, Israel; § Chemical Research Support, Weizmann Institute of Science, Rehovot 76100, Israel

## Abstract

Hydrogen bonds in molecular crystals are often modeled as double-well potentials, yet direct evidence linking this potential form to vibrational spectroscopic features remains elusive. In this study, we investigate α-glycine, a hydrogen-bonded crystal that exhibits pronounced Raman anomalies without undergoing a structural phase transition. Through temperature- and polarization-dependent Raman spectroscopy, supported by isotope substitution and first-principles calculations, we identify two peaks whose behavior violates conventional Raman selection rules. These peaks merge and narrow anomalously with temperature, an effect that cannot be explained by harmonic models or thermal broadening. Simulated spectra based on a weakly evolving asymmetric double-well potential reproduce this merging, indicating that both peaks originate from one double-well potential. Our results establish α-glycine as a model system directly linking microscopic hydrogen-bond potentials to vibrational spectroscopic features.

## Graphical abstract

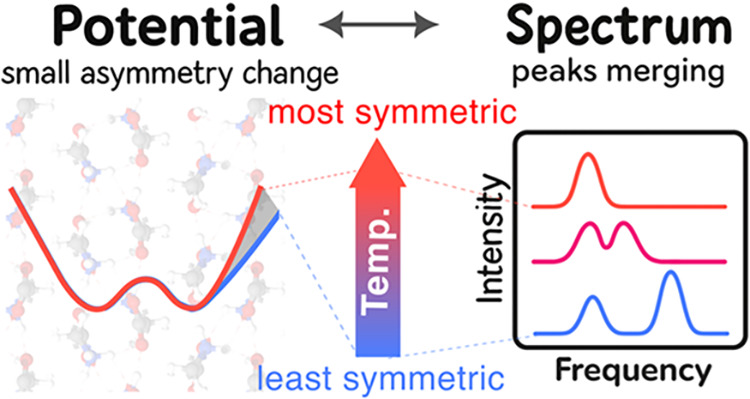

## Introduction

Hydrogen (H) bonds play a central role in determining the structural and dynamical behavior of a vast range of molecular crystals,^1–4^ from simple amino acids^5,6^ to complex biological assemblies.^7^ A H-bond is often modeled as an effective one-dimensional double-well potential energy surface,^2,8–11^ governing the proton motion between the hydrogen donor and acceptor. H-bonded crystals exhibit a variety of unconventional phenomena, including negative thermal expansion,^12–14^ ferroelectric phase transitions,^15^ unusually large mechanical stiffness,^16^ and sudden changes in spectroscopic^17^ and calorimetric^18^ response to temperature changes. These types of behavior are thought to be linked to the unique potential surface created by the H-bonds.

Among these systems, α-glycine is a prototypical example. Although it does not undergo a structural phase transition, α-glycine shows several anomalies upon temperature variations,^19–21^ including a change in dielectric constant,^22^ a change in the torsional motion of the amine group,^23–25^ and a distinct anomaly in its Raman spectra around 250 K, which was initially referred to as a “dynamical transition” or “molecular configurational change”.^17^ However, these observations lack a mechanistic framework that directly links the vibrational behavior to the underlying H-bond potential.

This raises a fundamental question: can the double-well potential characteristic of H-bonds be causally linked to specific vibrational features observed in H-bonded crystals? A mechanistic understanding of this connection would bridge the gap between microscopic interactions and macroscopic phenomena.

In this work, we establish a direct connection between the double-well potential of the H-bond and the vibrational behavior of α-glycine. Using polarization- and temperature-dependent Raman spectroscopy, we identify two peaks in the 490–520 cm^–1^ region that violate Raman selection rules expected from the average crystal symmetry, as determined by X-ray diffraction (XRD) and harmonic first-principles computation of the Raman spectrum. Isotope substitution confirms that these peaks are associated with H-bonds. The temperature evolution of the peaks is anomalous: they appear as distinct features at low temperatures but merge into a single asymmetric band as the temperature increases. Line shape analysis and phenomenological modeling show that this merging cannot be explained by thermal broadening or simple frequency shifts alone; it requires a temperature-dependent coupling between the modes. This increasing coupling introduces cross-correlation between the modes and suggests a thermally evolving anharmonic potential surface that governs their vibrational dynamics.

Finally, we present numerical simulations of spectral functions for vibrational modes in an asymmetric double-well potential, showing that two peaks can arise from such a potential and that even slight changes in the asymmetry substantially modify the spectral features. The simulations support the interpretation that both peaks originate from a single anharmonic potential, offering a qualitative mechanistic explanation for the temperature-dependent merging and isotope effects observed in our Raman experiments. Given that similar temperature-dependent vibrational behavior has been observed in other amino-acid crystals,^26–32^ this study establishes α-glycine as a model system that directly links microscopic H-bond potentials to vibrational spectroscopic signatures.

## Results and Discussion

Figure 1 highlights several anomalous features in the temperature evolution of the Raman spectra of an α-glycine single crystal (see Section S1 for materials and synthesis). Figure 1a depicts temperature-induced changes in the Raman spectra measured from 80 to 400 K. In the low-frequency region (<200 cm^–1^), associated with extended lattice vibrations, the phonon peaks exhibit redshifts and broadening as the temperature increases. These effects arise primarily from thermal expansion driven by the increasing phonon population, along with several previously reported anomalies attributed to subtle dynamical phenomena.^17^ Notably, the most temperature-sensitive feature is a pair of closely spaced peaks centered around 500 cm^–1^, highlighted by the gray-shaded region. Despite their relatively low thermal occupation (Bose–Einstein statistics predict populations ranging from only 0.1 to 9% for a mode at 500 cm^–1^, compared to 27 to 38% for a mode at 200 cm^–1^ over the same temperature range), these peaks evolve dramatically with temperature. While they are well resolved at low temperatures, they gradually coalesce into a single asymmetric feature with increasing temperature, displaying an unusual line shape transformation.

**Figure 1:**
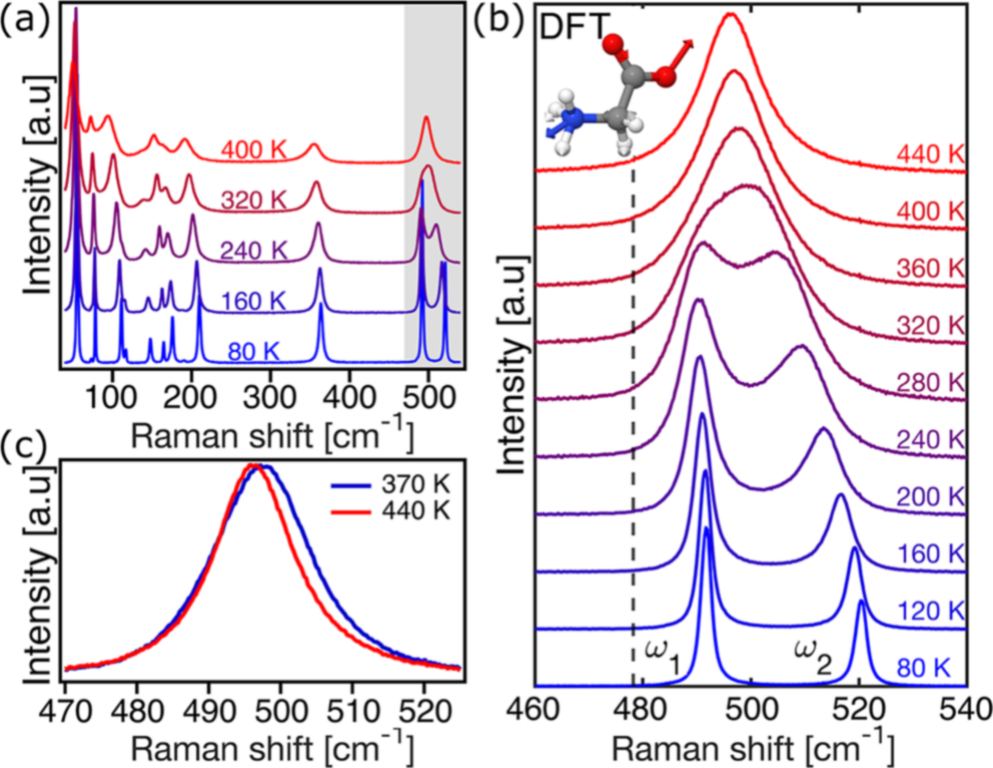
Temperature evolution of the Raman spectra: (a) Full range, from 40 to 550 cm^–1^, highlighting the low-frequency region (<200 cm^–1^) and the merging of two H-bond-related peaks (shaded in gray). (b) Magnified view of the evolution of the peaks in the shaded area of (a) between 80 and 440 K. The Dashed line shows the frequency of the mode calculated by DFT, accompanied by its eigenvector. Atom colors: red—oxygen, white—hydrogen, blue—nitrogen, gray—carbon. (c) Comparison of the merged peak at 370 K with the narrower spectrum at 440 K.

Figure 1b presents a magnified view of the gray-shaded region, tracking the merging of ω_1_ (492 cm^–1^, 80 K) and ω_2_ (520 cm^–1^, 80 K) into a single feature at 440 K. In the intermediate temperature range, the line shape appears non-Lorentzian: rather than a smooth broadening or simple merging, the spectrum assumes a complex shape that defies interpretation as two peaks merely expanding into each other. Notably, similar anomalous merging in this spectral region has been reported in other amino-acid crystals,^29,30,32^ including l-alanine^28,31^ and l-isoleucine.^27^ In α-glycine, however, the absence of overlapping vibrational features makes it particularly suitable for studying the temperature evolution of anomalous peak merging.

The assignment of ω_1_ and ω_2_ has been the subject of a longstanding debate. Early work by Machida et al.^33^ attributed ω_1_ to an NH_3_^+^ torsional vibration and ω_2_ to a COO^–^ rocking mode, based on normal coordinate analysis and isotopic substitution studies. This interpretation was supported by Murli et al.,^34,35^ who observed a splitting of the NH_3_^+^ torsional mode at low temperatures and under pressure, attributed to its strong sensitivity to intermolecular interactions. However, Stenback^36^ assigned ω_1_ to COO^–^ rocking but left ω_2_ unassigned, while Thaper et al.,^37^ using neutron scattering at 20 K, proposed the opposite assignment to Machida et al. and Murli et al., and also reported a splitting in the mode they identified as the NH_3_^+^ torsion. Chowdhry et al.,^38^ based on density functional theory (DFT) calculations, assigned ω_1_ to COO^–^ rocking and ω_2_ to CN torsion.

Similar to Chowdhry et al., our DFT calculations (see Figure 1b and Figure S1) also predict a single mode in this spectral region. We also included dispersion terms, which have proven effective for predicting Raman spectra of organic molecular crystals,^39,40^ to strengthen our confidence that the missing peak does not appear in the relevant spectral range (see Section S3 for computation details). Therefore, we find the assignment of ω_2_ to a CN torsional motion by Chowdhry et al. problematic, as the calculated frequency of ∼630 cm^–1^ significantly overestimates the experimental value. Instead, we propose that standard DFT calculations inherently yield only a single mode in this region. As we show below, including anharmonic effects with more complex vibrational potentials can generate two distinct peaks along the same normal coordinate, consistent with experimental observations. Indeed, a recent DFT study of glycine zwitterions in solution that included anharmonic corrections^41^ successfully reproduced both peaks in the Raman spectrum.

Another anomaly is illustrated in Figure 1c, which overlays the normalized spectra at 370 and 440 K. Since the sublimation point of α-glycine is 418 K,^42^ we used a custom-built cell apparatus, which minimizes sublimation effects, to reach higher temperatures (see Section S2 in the Supporting Information (SI) for details). Notably, the spectral feature narrows at higher temperatures, which is contrary to the typical broadening expected due to increased phonon–phonon scattering and reduced phonon lifetimes. This counterintuitive narrowing as the temperature increases is discussed below.

To further investigate the assignment of the two anomalous Raman peaks, we performed polarization–orientation (PO) measurements at 80 K, where the peaks are clearly distinct. Under the harmonic approximation, each Raman peak corresponds to a normal vibrational mode.^43,44^ The Raman response of each mode is characterized by a second-rank tensor determined by its eigenvector and the average crystal structure. Our PO measurements probe the polarization dependence of Raman scattering in both parallel and perpendicular configurations with respect to the incident polarization and allow extraction of the Raman tensors by fitting the angular intensity patterns of the scattered light (see Section S4 in the SI).^45,46^

For monoclinic α-glycine (space group *P*2_1_/*n*) observed from the (010) crystal face, only modes with an *A*_g_ irreducible representation are allowed, described by the following Raman tensor:^47^

$$A_{g}=\left(\begin{array}{lll} a & 0 & d\\ 0 & b & 0\\ d & 0 & c \end{array}\right)$$

Figure 2 illustrates the polarization-dependent intensity traces at the peak positions of ω_1_ and ω_2_, measured at 80 K, where the two peaks are clearly resolved. Filled circles represent experimental data in parallel (black) and perpendicular (red) polarization geometries. Solid lines show the highest-intensity traces extracted from a global fit to 146 PO spectra using a model consisting of two Lorentz oscillators, each described by a tensor of the form above and incorporating birefringence effects^48^ (see Section S4 in the SI for full PO maps and fitting details). The polarization dependence of ω_2_ is well-captured by this model. In contrast, the angular dependence of ω_1_ deviates from the second-rank Raman tensor description derived from the nominal structure, indicating that anharmonic effects contribute to the vibrational dynamics, giving rise to this peak. Notably, since all accessible tensor elements are nonzero in our geometry, extending the model to lower symmetry does not introduce additional degrees of freedom that would improve the fit.

**Figure 2:**
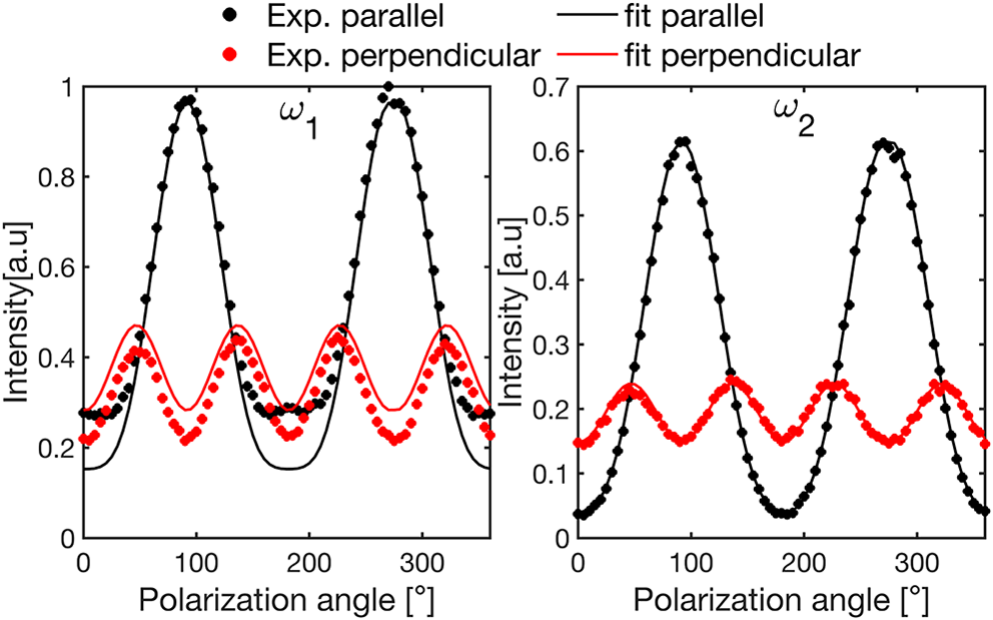
Polarization dependence of the maximum intensities of ω_1_ (left panel) and ω_2_ (right panel), at 80 K, shown for parallel (black) and perpendicular (red) polarization geometries. Filled circles represent experimental data, and solid lines correspond to a global fit of 146 spectra acquired in both parallel and perpendicular configurations.

To determine whether H-bonding affects the vibrational peaks ω_1_ and ω_2_, we compared the Raman spectra of α-glycine with that of N-deuterated α-glycine, in which only the H atoms participating in H-bonding were replaced with deuterium. H to deuterium substitution leaves the chemistry unchanged, though the altered nuclear mass in anharmonic potentials may reshape the vibrational landscape, suppress tunneling effects,^49^ and can in turn lead to subtle or significant spectral changes.^50,51^ The N-deuterated crystals were prepared via an exchange reaction with heavy water and grown under the same conditions as α-glycine but using D_2_O instead of H_2_O.^33^ Figure 3 overlays the Raman spectra of deuterated and nondeuterated α-glycine at 80 K (blue) and 300 K (red), measured from the (010) face, in the spectral range of ω_1_ and ω_2_. At 80 K, α-glycine shows two distinct peaks, while the N-deuterated sample exhibits a single asymmetric feature, consistent with previous reports.^52,53^ This region in the deuterated spectrum changes little between 80 and 300 K, unlike the merging of ω_1_ and ω_2_ in α-glycine, underscoring a strong temperature dependence linked to H-bond dynamics. Our DFT calculations of N-deuterated α-glycine also predict a single Raman mode with negligible shift relative to nondeuterated α-glycine (see Section S5 in the SI). These results indicate that H-bonds play a key role in shaping the temperature-dependent potential energy surface governing the spectral line shape.

**Figure 3:**
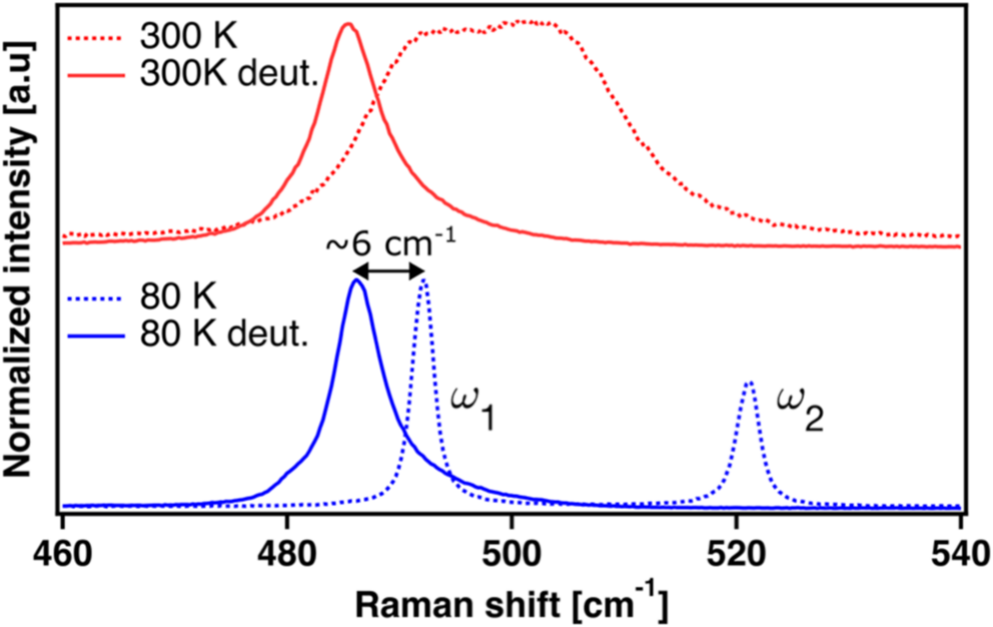
Normalized Raman spectra of α-glycine (dotted lines) and N-deuterated α-glycine (solid lines) at 80 K (blue, bottom) and 300 K (red, top). At 80 K, α-glycine exhibits two distinct peaks, ω_1_ and ω_2_, while the N-deuterated sample shows a single asymmetric feature. Upon heating to 300 K, the α-glycine spectrum changes significantly into a broad feature, whereas the spectrum of N-deuterated α-glycine remains nearly unchanged.

Having established that the appearance of ω_1_ and ω_2_ represents a violation of selection rules, associated with H-bonding, we return to the analysis of the abnormal temperature evolution of ω_1_ and ω_2_, including asymmetric merging and narrowing at high temperatures. While the most obvious explanation would have been a structural phase transition, α-glycine is known to remain structurally stable across a wide range of pressures and temperatures.^34,35,54,55^ Indeed, our temperature-dependent single-crystal XRD measurements, performed between 80 and 400 K, confirm the absence of any structural phase transitions. To investigate the possibility of subtle structural changes, we also performed a Hirshfeld atom refinement analysis of the XRD data (see Section S6 in the SI). However, no abnormalities were detected in the lattice parameters or positions of the H atoms. Nonetheless, numerous anomalies in the 80–440 K range have been reported, based on incoherent inelastic neutron scattering,^56^ nuclear magnetic resonance (NMR),^23,25,57^ dielectric constant measurements,^22^ and Raman spectroscopy.^17^ In particular, infrared spectroscopy measurements^52^ revealed anharmonic temperature trends near 80 and 210 K in both the frequency and intensity of the same peaks investigated in this study.

We hypothesize that the unusual temperature evolution of ω_1_ and ω_2_ originates from nontrivial vibrational anharmonicity. To test this, we fit the temperature-dependent Raman spectra using a Green’s function–based model that enables systematic variation of anharmonic contributions (for full model and fitting details, see Section S7 in the SI). The retarded Green’s function is constructed as the sum of a harmonic Green’s function, *g*_0_, and a self-energy matrix, Σ, which encode the harmonic and anharmonic contributions, respectively. For our two-mode system, the harmonic Green’s function and self-energy take the form:

1 $$g^{0}\left(\Omega \right)=\left(\begin{array}{ll} \frac{2\omega_{1}}{\Omega^{2}-\omega_{1}^{2}} & 0\\ 0 & \frac{2\omega_{2}}{\Omega^{2}-\omega_{2}^{2}} \end{array}\right),\Sigma \left(\Omega \right)=\left(\begin{array}{ll} i\Gamma_{1} & \gamma \\ \gamma & i\Gamma_{2} \end{array}\right)$$

where Ω is the excitation energy, ω_1_ and ω_2_ are the mode frequencies, Γ_1_ and Γ_2_ are the inverse lifetimes, and γ is a complex off-diagonal term that couples the two modes. We first consider the uncoupled case, γ = 0, in which Green’s function remains diagonal and the spectrum consists of two independent Lorentzian peaks. The fit results are shown in Figure 4a–c (red curves) for three representative temperatures, plotted on a semilogarithmic scale (fits at all temperatures are shown in Figures S8 and S9 in the SI in semilog and linear scales, respectively). The spectra were obtained from polarized Raman measurements in a fixed configuration and corrected by a Bose–Einstein factor. While the model reproduces the data reasonably well at 80 K, it fails at higher temperatures, overestimating the edges and underestimating the dip between the peaks.

**Figure 4:**
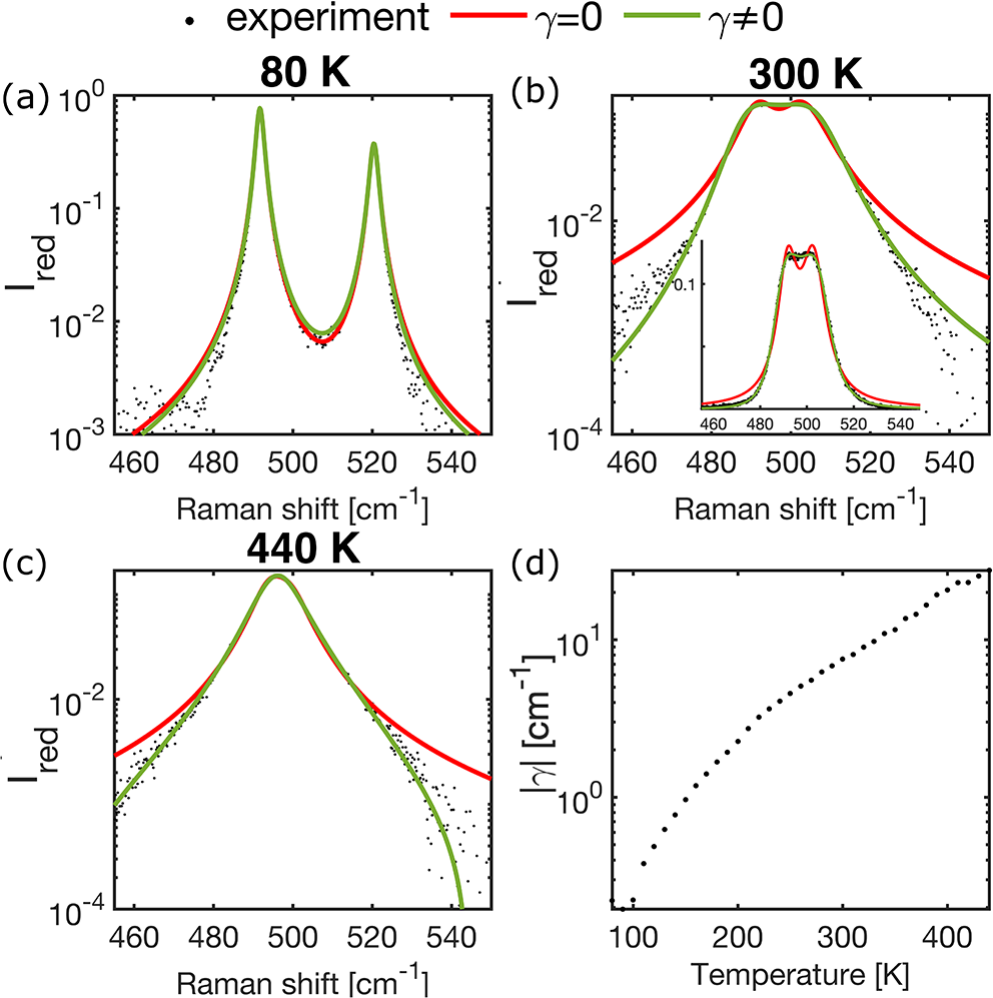
(a–c) Experimental spectra at 80, 300, and 440 K, respectively, reduced by the Bose–Einstein occupation factor (black dots). Spectra are presented as semilog plots for clarity; inset in (b) is in a linear scale. Red and green curves show the fit to two uncoupled Lorentz oscillators (two Lorentzians) and two coupled Lorentz oscillators, respectively. (d) Temperature evolution of the specific coupling of the two modes on a semilog plot, demonstrating that the cross-correlation between the modes increases with temperature. For the evolution of all fitting parameters, refer to Figure S10.

Next, we allow γ ≠ 0 in eq 1, introducing an off-diagonal self-energy that couples ω_1_ and ω_2_. This coupling generates temperature-dependent cross-correlations between the modes, breaking the normal-mode picture and yielding asymmetric, non-Lorentzian lineshapes. This coupled-mode approach has been previously used in several cases to model asymmetric lineshapes.^58–65^ The coupled-mode fits, represented by the green curves in Figure 4a–c and Figures S8 and S9, accurately capture the full temperature dependence. The corresponding fitting parameters are physically reasonable, as they evolve continuously with temperature, and the magnitudes of all of the parameters in the self-energy matrix are comparable. To rule out instrumental effects, we fit the spectra with two pseudo-Voigt profiles (see Section S7 in the SI), which yield slightly improved fits over the Lorentzian model but remain inaccurate. Given that the coupled-mode model provides a better overall fit using fewer fitting parameters, we conclude that it is the most appropriate description.

Figure 4d shows that cross-correlation between the modes increases with temperature, as demonstrated by the temperature dependence of the coupling constant γ. The temperature evolution of the other fitting parameters is shown in Figure S10 in the SI. At low temperatures, the coupling remains negligible, consistent with the adequacy of the independent Lorentzian description. However, as the temperature increases, the cross-correlation between the modes grows continuously, signaling a temperature-driven evolution of the anharmonic potential landscape. Importantly, the temperature dependence of the coupling constant is nonlinear, showing distinct trends: it begins to increase logarithmically near 100 K and deviates from this behavior between 210 and 240 K. These temperature intervals coincide with those of previously reported anomalies in α-glycine,^17,22,23,25,52,56,57^ as discussed earlier.

Lastly, we propose a microscopic mechanism to account for the anomalous observations. The polarization dependence, isotope substitution, and temperature evolution of the Raman spectra all indicate that the ω_1_ and ω_2_ peaks in α-glycine violate Raman selection rules and originate from an anharmonic potential energy surface that evolves with temperature. The simplest model yielding two peaks that merge as the temperature varies is an asymmetric double-well potential that becomes progressively more symmetric as the temperature increases. We illustrate this evolution using a simplified one-dimensional simulation, where the double-well asymmetry is controlled by a parameter *a*. The potential is constructed as a symmetric double-well plus a linear term weighed by *a*, with *a* = 1 corresponding to the most asymmetric case and *a* = 0 to a fully symmetric double-well, as shown in Figure S14 in the SI. Simulation details are provided in Section S8 of the SI. Notably, to fully reproduce the polarization-orientation result, a model of a three-dimensional asymmetric double-well potential with corresponding susceptibility tensors is required, which is beyond the scope of this study. Also, other double-well scenarios such as proton tunneling or varying well depths are possible,^50^ yet more complicated, and were outside the scope of this work.

Figure 5a illustrates the two limiting cases of the double-well potential considered, corresponding to low temperature (blue, *a* = 1) and high temperature (red, *a* = 0.4). Figure 5b shows the simulated spectral function evolution between the corresponding potentials in steps of *a* = 0.1, qualitatively reproducing the merging of ω_1_ and ω_2_ with increasing temperature. To simulate the spectral function, for each potential, we computed the eigenfunctions ψ_*j*_ and eigenvalues *E*_*j*_ and evaluated the squared position matrix elements |⟨ψ_*f*_ | *x* |ψ_*i*_⟩|^2^, where ψ_*i*_ and ψ_*f*_ are the initial and final eigenfunctions, respectively. The initial-state populations were fixed, assuming negligible population variation across the measured temperature range. At these temperatures, only low-lying states are significantly populated, and all spectral features arise from excitations below the barrier (Figure S15 in the SI). Transitions between such nearly degenerate pairs (e.g., [0,1], [2,3]) were excluded, as they vanish in the symmetric limit and do not give rise to this peak splitting. Therefore, for relatively symmetric potentials (small *a*), the low-energy eigenstates are nearly degenerate, producing a single peak. As asymmetry increases, the degeneracy is lifted and two distinct peaks emerge. Figure 5c reproduces the anomalous spectral narrowing observed experimentally between 370–440 K in Figure 1c. As the potential becomes more symmetric (i.e., smaller *a*), the eigenenergies of adjacent states converge and the resulting spectral feature narrows. The double-well framework thus naturally explains the observed narrowing with increasing temperature.

**Figure 5:**
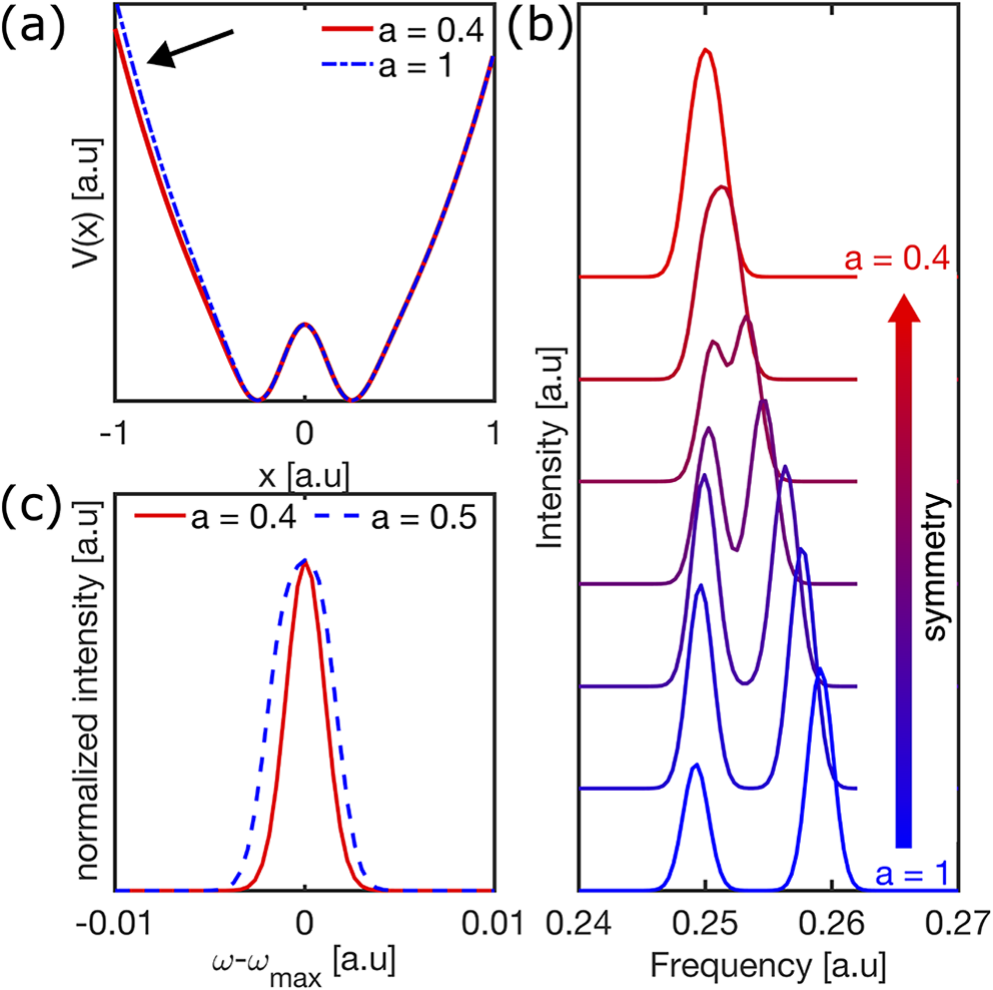
(a) Comparison of two double-well potentials with asymmetry coefficients *a* = 1 (blue) and *a* = 0.4 (red). (b) Simulated spectral function corresponding to double-well potentials with asymmetry coefficients decreasing from *a* = 1 to *a* = 0.4 in steps of 0.1. At *a* = 1, the asymmetric potential (bottom, blue) produces two distinct peaks. These progressively merge into a single feature as the potential becomes more symmetric. All parameters are expressed in arbitrary units. (c) Comparison of the spectral functions for *a* = 0.5 (blue) and *a* = 0.4 (red), aligned at their maximum intensities, demonstrating spectral narrowing as the double-well symmetry increases.

In this one-dimensional model, the parameter *a* quantifies the projection of many-body forces onto the double-well asymmetry, showing that even modest variations can induce significant spectral shifts, accounting for the pronounced temperature sensitivity of the ω_1_ and ω_2_ line shape. Since the temperature evolution of double-well potentials often leads to critical behavior, determining *a* as a function of temperature is particularly important. In the present work, the model is used to capture spectral trends analogous to the experimental results, rather than to reproduce the spectra directly. In future studies, we aim to reformulate the model for direct fitting to Raman data, enabling rigorous testing of whether *a* exhibits critical behavior and allowing its temperature dependence to be explored across phase transitions in other materials.

## Conclusions

This work demonstrated that the vibrational behavior of α-glycine is governed by a thermally evolving anharmonic potential, specifically modeled as an asymmetric double well. We suggest that the violation of the Raman selection rules, anomalous line shape merging, and isotope sensitivity observed in the ω_1_ and ω_2_ peaks are rooted in the characteristics of this anharmonic potential. Computational simulation shows that modest variations in asymmetry can lead to significant spectral changes, resembling the experimental temperature dependence. These results highlight the critical role of H-bond dynamics in shaping vibrational spectra, even in systems with no apparent structural transitions. Overall, this study provides a quantitative framework for linking spectroscopic anomalies to microscopic potential surfaces in H-bonded crystals.

## Supplementary Material









## Notes

Materials and experimental details, DFT computation details of Raman modes including isotope substitution, polarization-orientation analysis, single-crystal XRD analysis, temperature-dependent fits and analysis, and numerical simulation details (PDF)
Calculated vibrational frequencies of α-glycine and N-deuterated α-glycine (XLSX)
DFT calculated eigenvector along *a*–*b* plane (MP4)
DFT calculated eigenvector along *b*–*c* plane (MP4)
