# Supplementary material for: Hydrogen Bonds Induce Double-Well Spectroscopic Signatures in α‑Glycine
Source: J Am Chem Soc. 2025 Oct 31;147(45):41699–705. doi: 10.1021/jacs.5c13223 (PMC12616690; doi:10.1021/jacs.5c13223)
Supplement: Supplementary file 1 [file ja5c13223_si_001.pdf]

Hydrogen Bonds Induce Double-Well Spectroscopic Signatures in α-glycine

Noam Pinsk,[1] Nimrod Benshalom,[1] Michal Hartstein,[2] Yael Diskin-Posner,[3]
Matan Menahem,[1] Olle Hellman,[2] Leeor Kronik,[2] and Omer Yaffe[1,*]

[1]*Department of Chemical and Biological Physics,*
Weizmann Institute of Science, Rehovot 76100, Israel
[2]*Department of Molecular Chemistry and Materials Science,*
Weizmann Institute of Science, Rehovot 76100, Israel
[3]*Chemical Research Support, Weizmann Institute of Science, Rehovot 76100, Israel*

[*] omer.yaffe@weizmann.ac.il

S1. Materials and synthesis

Glycine 99% purity was purchased from Alfa Aesar and dissolved in hot double distilled water (DDW) at a ratio of 30 g powder to 100 mL DDW. α-glycine was grown by slow evaporation of the saturated solution. Harvested crystals were annealed at 100°C for at least an hour to release the water trapped at the surface[1]. For extra dehydration the α-glycine crystals were left overnight in vacuum (10^{-4} Torr). Deuterated-α-glycine was prepared by the same procedure except the DDW was switched with D_2O.

S2. Temperature dependent and polarization-orientation Raman measurements

Raman measurements were performed using a custom-built dispersive Raman spectrometer based on a 1-meter Horiba FHR-1000 system. The setup included notch filters that allowed access to the low-frequency Raman region down to ~ 10 cm^{-1}. Excitation was provided by a Prometheus 532 nm Nd:YAG laser, with the laser power on the sample maintained at approximately 9 mW.

The detection system included a $10\times$ objective and a 2400 mm^{-1} grating, yielding a spectral resolution of ~ 0.27 cm^{-1}. Control over the polarization of both the incident and scattered light was achieved using half-wave plates in combination with a polarizer–analyzer setup, as previously described in Ref.[2].

Temperature control was provided by a Janis ST-500 cryostat equipped with a Lakeshore 335 temperature controller. Temperature-dependent measurements were carried out in steps of 10 K between 80K and 440K. A polarized spectrum at a constant angle with respect to the crystal axis was collected at each temperature. To prevent extreme sublimation of the sample when held above the sublimation temperature, the α-glycine crystals were placed in a small sealed cell filled with Helium gas to ensure atmospheric pressure, and saturation of the glycine gas within the cell that suppresses further sublimation of the sample.

Polarization-orientation (PO) measurements were performed at 80 K. A total of 146 spectra were acquired by rotating the incident polarization from 0° to 360° in 5° increments, using a half-wave plate. For each incident polarization angle, spectra were recorded in two detection configurations: parallel (0°) and perpendicular (90°) with respect to the incident polarization.

S3. Computational methods

All calculations were performed within the framework of DFT, using the Vienna ab initio simulation package (VASP) version 6.3.0 [3]. All calculations were performed using the Perdew-Burke-Ernzerhof (PBE) exchange correlation functional[4], augmented by D3 dispersive terms with the Becke-Johnson damping function[5,6]. As a first step, full atomic relaxations were performed, starting from the experimentally measured structure of α-glycine[7], with a threshold of 10^{-3} eV $\mathring{A}^{-1}$ for the residual forces, a k-point grid sampling of the Brillouin zone of 3×2×3, and a plane-wave cutoff of 1000 eV. Finite differences with symmetry, in the harmonic approximation, were used to calculate phonon frequencies, with an atomic displacement of 0.01 Å. In order to evaluate the effect of including the non-analytical-correction (NAC) term on the resulting frequencies, phonon modes were also calculated using the Phonopy simulation package[8,9] and compared to those generated with VASP only. Born effective charges were calculated using a double k-point grid of 6×4×6 and then used to calculate the non-analytical term, which was added to the dynamical matrix calculated by Phonopy to obtain the phonon modes. Those were very similar to those calculated without NAC, with small differences of up to 2.3 cm^{-1} for modes below 700 cm^{-1}. For the monoclinic $P2_1/n$ space group, only the gerade representations A_g and B_g are Raman active. However, only A_g modes were considered for the spectrua simulation as B_g modes were not accessible experimentally (see discussion in main text).

Figure S1 shows the eigenmode of the calculated A_g mode at 477.68 cm^{-1}, on four molecules along the b–c plane (left panel) and the a–b plane (right panel).

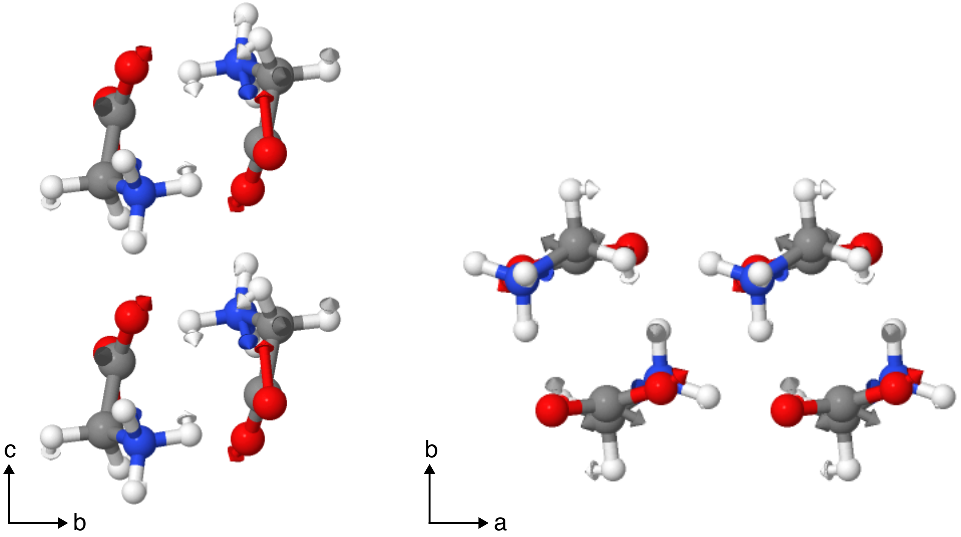

Figure S1: Calculated eigenmode along the b–c and a–b planes in the left and right panels respectively.

S4. Polarization-orientation analysis

Figure S2 presents the polarization–orientation (PO) Raman data of α-glycine in the spectral range 480–540 cm^{-1}, acquired at 80 K and reduced by the Bose–Einstein occupation factor. This reduction was performed by dividing the spectra by $(n_{\mathrm{BE}}+1) = (\frac{1}{\exp(h\Omega/k_B T)-1}+1)$, where h is Planck's constant, Ω is the excitation energy, k_B is Boltzmann's constant, and $T = 80$ K is the measurement temperature. The left and right panels show the parallel and perpendicular scattered polarization geometries relative to the incident polarization. Each map consists of 73 spectra recorded in 5° steps of the incident polarization angle, totaling in 146 spectra. White dashed lines indicate the positions of the maximum-intensity as a function of polarization angle, which are shown in Figure 1d.

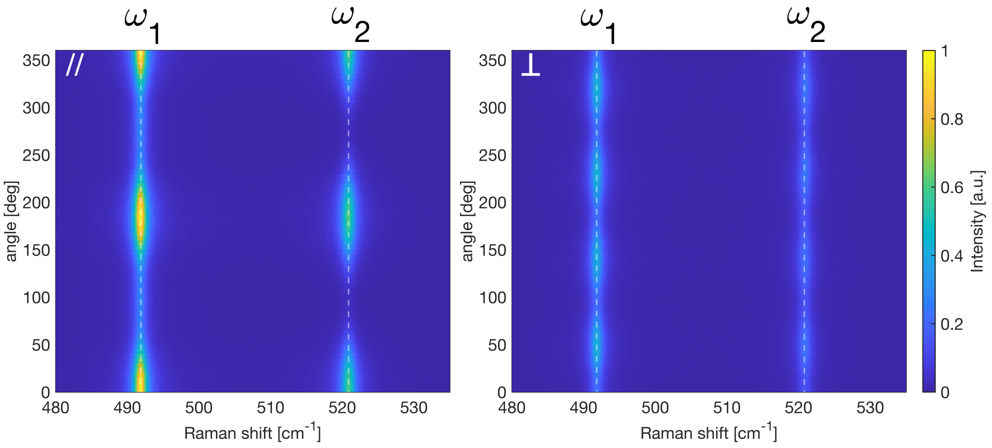

Figure S2: Polarization–orientation (PO) maps of ω_1 and ω_2 at 80 K, recorded in parallel (left) and perpendicular (right) polarization geometries. White dashed lines mark the highest-intensity traces shown in Figure 1d.

To extract the Raman tensors of ω_1 and ω_2, we performed a simultaneous global fit to the complete PO dataset shown in Figure S2, comprising 146 spectra collected in both parallel and perpendicular polarization configurations. Below, we derive the angular dependence of the Raman response using group theory, incorporating both the crystal symmetry and the measurement geometry.

At fixed temperature, the Raman scattering cross section for a normal mode transforming as Γ_λ irreducible representation is given by[10]:

$$\sigma_{\Gamma_\lambda} \propto \left| \hat{\vec{e}}_i \cdot \mathcal{R}_{\Gamma_\lambda} \cdot \hat{\vec{e}}_s \right|^2, \tag{S1}$$

where $\hat{\vec{e}}_i$ and $\hat{\vec{e}}_s$ are the unit vectors of the incident and scattered polarizations, respectively, and $\mathcal{R}_{\Gamma_\lambda}$ is the Raman tensor. In our backscattering configuration on the (010) face, only the x/z components contribute, yielding:

$$\hat{\vec{e}}_i = \begin{pmatrix} \cos(\theta - \theta_0) \\ 0 \\ \sin(\theta - \theta_0) \end{pmatrix}, \tag{S2}$$

where θ is the incident polarization angle and θ_0 is the arbitrary offset between the crystal and laboratory frames. In the parallel configuration, the incident and scattered polarizations are the same, $\hat{\vec{e}}_s = \hat{\vec{e}}_i$. In the perpendicular configuration, the scattered polarization is orthogonal to the incident polarization, giving:

$$\hat{\vec{e}}_s = \begin{pmatrix} -\sin(\theta - \theta_0) \\ 0 \\ \cos(\theta - \theta_0) \end{pmatrix}. \tag{S3}$$

To account for optical birefringence, we introduce a Jones matrix J[11], modifying Eq. (S1) as:

$$\sigma_{\Gamma_\lambda}(\theta;\theta_0) \propto \left| \hat{\vec{e}}_i \cdot J\mathcal{R}_{\Gamma_\lambda} J \cdot \hat{\vec{e}}_s \right|^2, \tag{S4}$$

where

$$J = \begin{pmatrix} 1 & 0 & 0 \\ 0 & 0 & 0 \\ 0 & 0 & e^{i\delta} \end{pmatrix}, \tag{S5}$$

and δ is a birefringent phase shift treated as a global fit parameter. In monoclinic α-glycine (space group $P2_1/n$), only A_g modes are symmetry-allowed under this geometry[12], with the corresponding Raman tensor:

$$\mathcal{R}_{A_g} = \begin{pmatrix} a & 0 & d \\ 0 & b & 0 \\ d & 0 & c \end{pmatrix}.$$

The Stokes Raman intensity of mode λ, as a function of Raman shift Ω and polarization angle θ, at fixed temperature T and offset θ_0, is given by:

$$I_\lambda(\Omega,\theta;T,\theta_0) = [n(\Omega;T)+1]\,\sigma_{A_g,\lambda}(\theta;\theta_0)\,J_\lambda(\Omega;T), \tag{S6}$$

where $n(\Omega;T)$ is the Bose–Einstein distribution, and $J_\lambda(\Omega;T)$ is a Lorentzian spectral function:

$$J_\lambda(\Omega;T) = \frac{4\omega_\lambda|\Omega|\Gamma_\lambda}{(\Omega^2-\omega_\lambda^2)^2+4\Gamma_\lambda^2\Omega^2}, \tag{S7}$$

with ω_λ and Γ_λ denoting the mode frequency and linewidth (HWHM), respectively. Removing the Bose factor from Eq. (S6) yields the reduced Raman intensity, used for fitting. For the parallel configuration:

$$\begin{aligned} I_{red}^{\lambda,\parallel}(\Omega,\theta) = &\left|a_\lambda\cos^2(\theta-\theta_0)+2d_\lambda\sin(\theta-\theta_0)\cos(\theta-\theta_0)+c_\lambda e^{i\delta}\sin^2(\theta-\theta_0)\right|^2 \\ &\cdot\frac{4\omega_\lambda|\Omega|\Gamma_\lambda}{(\Omega^2-\omega_\lambda^2)^2+4\Gamma_\lambda^2\Omega^2} \end{aligned} \tag{S8}$$

and for the perpendicular configuration:

$$\begin{aligned} I_{red}^{\lambda,\perp}(\Omega,\theta) = &\Big|-a_\lambda\sin(\theta-\theta_0)\cos(\theta-\theta_0)+d_\lambda\left[\cos^2(\theta-\theta_0)-\sin^2(\theta-\theta_0)\right] \\ &+c_\lambda e^{i\delta}\sin(\theta-\theta_0)\cos(\theta-\theta_0)\Big|^2\cdot\frac{4\omega_\lambda|\Omega|\Gamma_\lambda}{(\Omega^2-\omega_\lambda^2)^2+4\Gamma_\lambda^2\Omega^2} \end{aligned} \tag{S9}$$

These expressions were used to simultaneously fit all 146 reduced PO spectra as functions of Raman shift Ω and polarization angle θ, allowing extraction of the Raman tensor elements a_λ, c_λ, and d_λ, along with the mode parameters ω_λ and Γ_λ for both modes.

The resulting fitting parameters are shown in table S1:

ω_1	Γ_1	a_1	c_1	d_1	ω_2	Γ_2	a_2	c_2	d_2	θ_0	δ
491.7 cm^{-1}	0.83 cm^{-1}	−0.89	−2.25	1.22	520.7 cm^{-1}	0.91 cm^{-1}	0.46	1.86	0.92	91.86°	1.33 [rad]

Table S1: Fitting parameters obtained from the model.

Figure S3 shows the resulting PO map based on these fitting parameters. In Figure 1d in the main text, we compare the fit with the experimental data by overlaying the highest-intensity angular traces of ω_1 and ω_2. The fit shows excellent agreement for ω_2, while ω_1

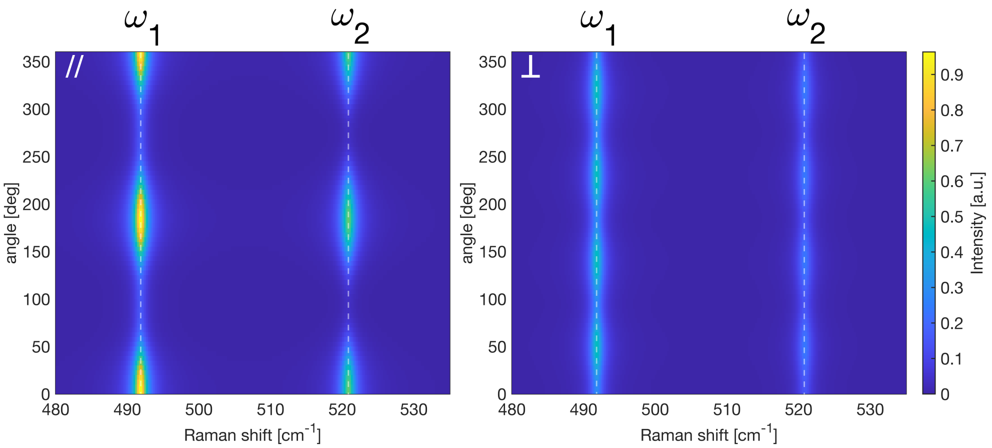

Figure S3: Polarization–orientation (PO) maps of the global fit to the PO map in Figure S2 parallel (left) and perpendicular (right) polarization geometries. White dashed lines mark the highest-intensity traces shown in Figure 1d.

exhibits a clear deviation from the expected model.

S5. Isotope exchange analysis

N-deuterated α-glycine was prepared using the same procedure as non-deuterated α-glycine (see Experimental Section in the main text), except that DDW was replaced with D_2O. Correspondingly, Density Functional Theory (DFT) calculations were based on the same α-glycine crystal structure, with only the hydrogen atoms involved in hydrogen bonding replaced by deuterium. A single A_g Raman mode was identified in the corresponding spectral region, with negligible frequency shift from the calculated mode in non-deuterated α-glycine. Table S2 presents the DFT-calculated frequencies of the A_g modes for both α-glycine and N-deuterated α-glycine in the experimental spectral range.

Table S2: Comparison between the A_g mode frequencies in α-glycine and N-deuterated α-glycine in the measured spectral range

	**α-glycine**	**N-deuterated α-glycine**	**Δ**
A_g **Frequency [cm^{-1}]**	477.64	477.78	0.14

S6. Single crystal X-Ray Diffraction

Data were collected from a single crystal. The crystal was glued with a small amount of nail polish to a broken Mitogen loop. See Fig. S4. The crystal was mounted at room temperature and was cooled/heated from room temperature to the designated measurement

temperature at the rate of 1 K/min. When the designated measurement temperature was reached, the crystal was allowed to settle for an additional 5 minutes. A data collection strategy was calculated to measure a high-resolution, complete, and redundant dataset with AgKα (λ=0.56087 Å). Before each dataset measurement, a movie of the crystal was recorded.

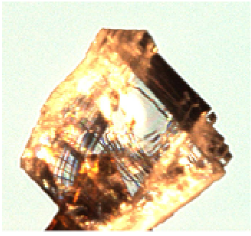

Figure S4: The mounted crystal used for data collection.

Data were collected on a Rigaku Synergy-S diffractometer dual source equipped with a Dectris Pilatus3 R CdTe 300K detector and a microfocus, with ω scans. This diffractometer is equipped with a Pilatus detector, which is specifically highly sensitive to AgKα radiation. The data collection strategy was devised for a redundant and high-resolution publishable structure. Data were integrated with CrysAlisPRO, and a Gaussian absorption correction was applied. Data were collected at 80 K, 200 K, 220 K, 240 K, 260 K, 280 K, 300 K, 380 K, 390 K, and 400 K, integrated and processed according to the highest and most suitable Laue symmetry.

The data resolution was cut to make sure that the $I/\sigma(I)>3$, even in the highest resolution bin. Structures were solved using SHELXT[13] and further refined with SHELXL[14] by full matrix least-squares refinement based on F^2. SHELXL is incorporated in the OLEX2[15] GUI platform. Structure was further refined by using the NoSpherA2[16] Module. The program NoSpherA2 is an implementation of the Hirshfeld Atom Refinement (HAR). This refinement makes use of tailor-made aspherical atomic form factors calculated on-the-fly from a Hirshfeld-partitioned electron density (ED). Unlike the SHELXL refinement which is using spherical-atom form factors.

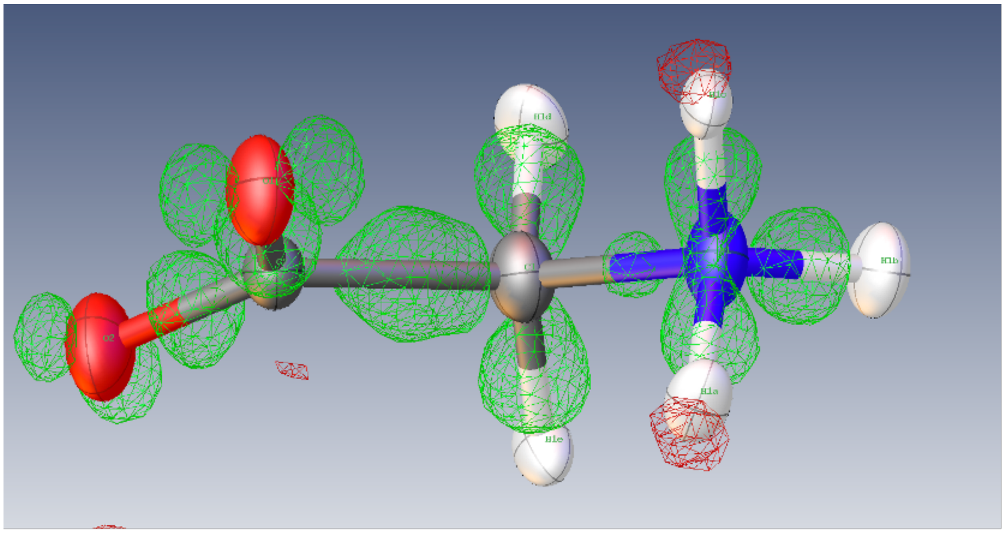

Figure S5: ORTEP presentation of the refined glycine molecule. The green mesh is the calculated electron density, concentrated along the bonds and around the lone electron pairs. The red mesh indicates a slight withdrawal of electron density from the hydrogen atoms, exposing them to hydrogen bonding.

The ED is calculated from a Gaussian basis set single determinant SCF wavefunction, either by Hartree-Fock or by DFT using selected functionals. This is done for a fragment of the crystal. This fragment can be embedded in an electrostatic crystal field by employing cluster charges or modeled using implicit solvation models, depending on the software used. We used the ORCA 5.0 software, the method chosen is r2SCAN, and the basis set used is def2-TZVPP. Refinement took into account the three closest neighbors, which generate hydrogen bonds. See Fig. S5, S6. The green mesh is the refined electron density map. Red mesh presents a withdrawal of electron density. Crystallographic data is summarized in table S3. Figure S7 shows the lattice parameters extracted at 80 K, 200–300 K, and 380–400 K. The black data points represent averages over four single crystals, with error bars indicating the standard deviation. The red data point corresponds to a single crystal, with error bars omitted since the measurement error is small compared to the standard deviation of the averaged data. Between 200-300 K, the lattice parameters evolve continuously with temperature, with thermal expansion along the a and b axes, while the c axis remains constant within error. This behavior persists outside this range, with no indication of a structural phase transition.

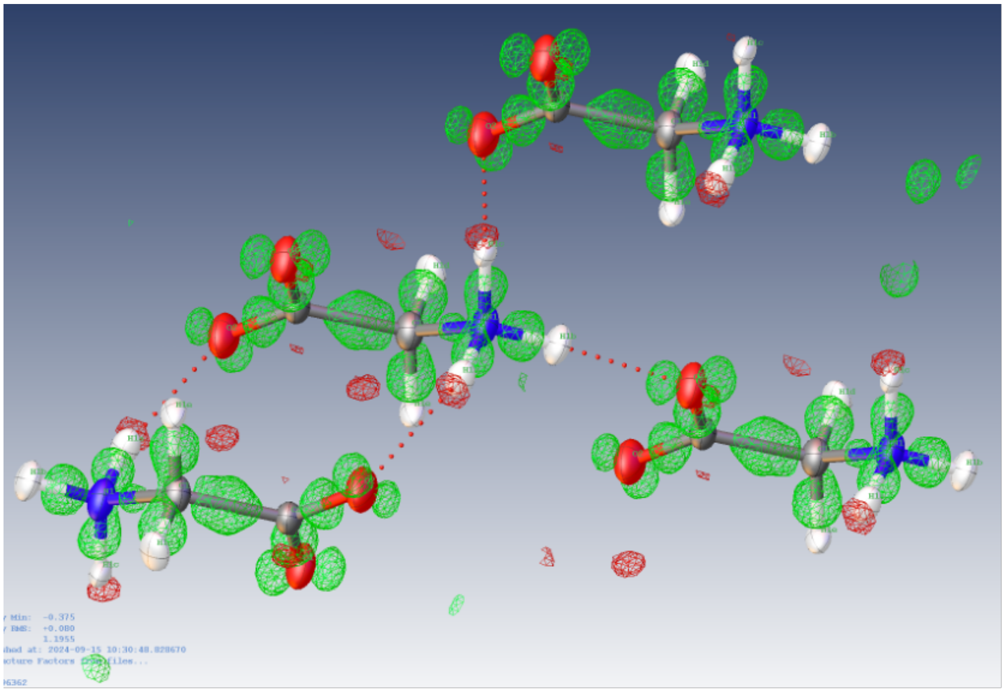

Figure S6: ORTEP presentation of the glycine molecule with its three hydrogen-bonded neighbors. Red dashed lines indicate hydrogen bonds. The green mesh shows the refined electron density; the red mesh highlights electron density withdrawal from the hydrogen atoms.

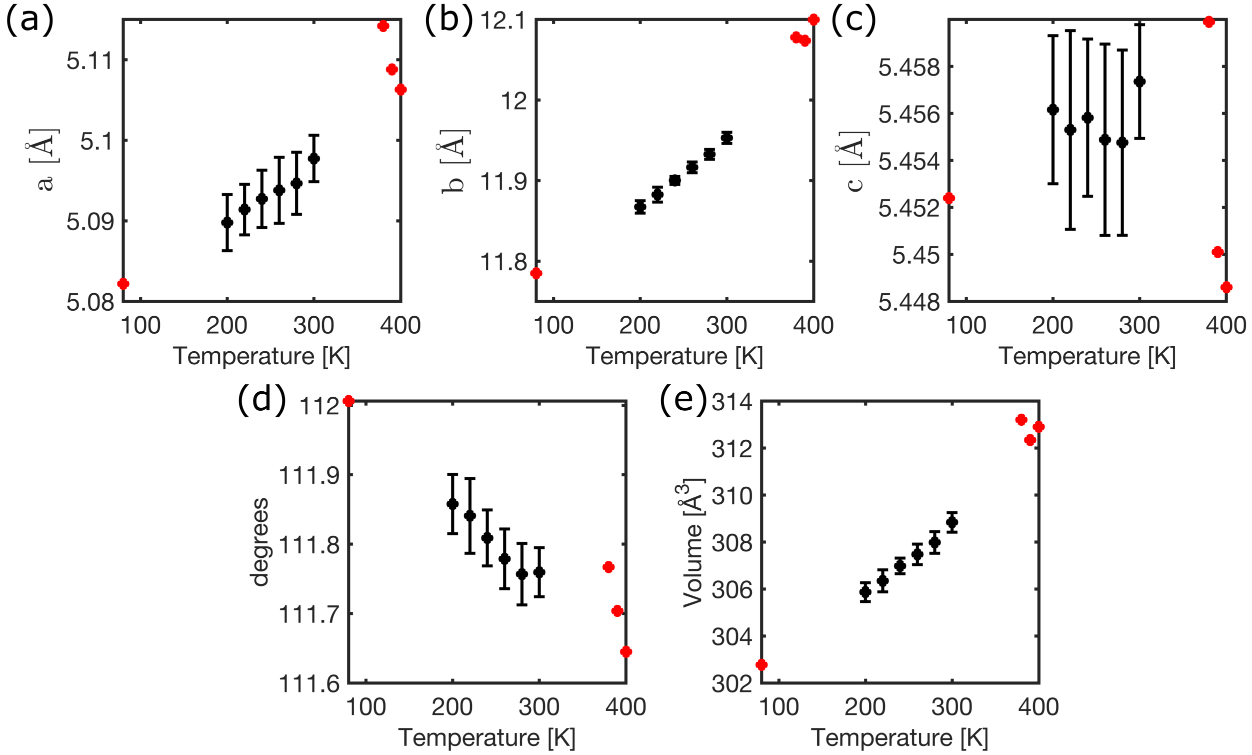

Figure S7: Lattice parameters as a function of temperature. Black data points and error bars indicate an average over four different single crystals. Red data points represent data at 80 K, 380 K, 390 K,400 K from a single crystal. Error bars were omitted because the errors are smaller than the common standard deviation.

Table S3: Crystallographic Data for $C_2H_5NO_2$ at Various Temperatures (Part 1 of 2)

Temperature (K)	80	200	220	240	260	280
CCDC No.	2469545	2469598	2469600	2469599	2469601	2469602
Wavelength (Å)	0.56087					
Formula	$C_2H_5NO_2$					
Formula weight	75.068					
Crystal system	Monoclinic					
Space group	$P2_1/n$					
Crystal size (mm)	0.67×0.24×0.21	0.69×0.26×0.23				
Crystal color and shape	Yellow, prism					
a (Å)	5.0822	5.0891	5.0900	5.0911	5.0944	5.0953
b (Å)	11.7850	11.8632	11.8784	11.8920	11.9144	11.9303
c (Å)	5.4524	5.4577	5.4583	5.4587	5.4586	5.4586
α (°)	90					
β (°)	112.006	111.827	111.808	111.784	111.789	111.765
γ (°)	90					
Volume (Å^3)	302.773	305.88	306.40	306.89	307.65	308.17
Z	4					
Density (g/cm^3)	1.647	1.630	1.627	1.625	1.621	1.618
μ (mm^{-1})	0.088	0.087	0.087	0.087	0.086	0.086
h range	-12 to 7	-10 to 5				
k range	0 to 29	0 to 23				
l range	-6 to 13	-5 to 10				
No. reflections (unique)	59952 (4883)	22244 (2563)	22270 (2566)	22163 (2569)	22805 (2574)	22859 (2579)
θ_{max} (°)	44.33	34.11	34.11	34.11	34.11	34.11
R_{int}	0.0480	0.0322	0.0322	0.0320	0.0290	0.0274
Completeness (%)	99.61	99.96	99.96	99.96	99.96	99.96
Data/restraints/parameters	4883/0/91	2563/0/91	2566/0/91	2569/0/91	2574/0/91	2579/0/91
Goodness-of-fit on F^2	0.8680	0.9281	0.9952	0.9489	0.9539	1.0423
R_1 [I>2σ(I)]	0.0212	0.0179	0.0181	0.0195	0.0209	0.0214
wR_2 [I>2σ(I)]	0.0469	0.0406	0.0405	0.0430	0.0507	0.0519
R_1 (all data)	0.0270	0.0213	0.0220	0.0236	0.0255	0.0263
wR_2 (all data)	0.0490	0.0418	0.0418	0.0445	0.0528	0.0535

Table S3: (continued)

Temperature (K)	300	380	390	400
CCDC No.	2469603	2469548	2469547	2469546
Wavelength (Å)		0.56087		
Formula		$C_2H_5NO_2$		
Formula weight		75.068		
Crystal system		Monoclinic		
Space group		$P2_1/n$		
Crystal size (mm)	0.69×0.26×0.23	0.49×0.18×0.16	0.46×0.20×0.16	
Crystal color and shape		Yellow, prism		
a (Å)	5.0950	5.1142	5.1088	5.1063
b (Å)	11.9547	12.0779	12.0736	12.0998
c (Å)	5.4580	5.4599	5.4501	5.4486
α (°)		90		
β (°)	111.733	111.767	111.704	111.645
γ (°)		90		
Volume (Å^3)	308.81	313.21	312.34	312.91
Z		4		
Density (g/cm^3)	1.615	1.592	1.596	1.593
μ (mm^{-1})	0.086	0.085	0.085	0.085
h range	-10 to 5	-8 to 4	-8 to 4	
k range	0 to 23	0 to 19	0 to 19	0 to 18
l range	-5 to 10	-4 to 8	-4 to 8	-4 to 8
No. reflections (unique)	22915 (2581)	9954 (2286)	7564 (1370)	7045 (1313)
θ_{max} (°)	34.11	26.43	26.89	26.43
R_{int}	0.0273	0.0782	0.0332	0.0294
Completeness (%)	99.96	99.39	99.93	99.93
Data/restraints/parameters	2581/0/91	1303/0/91	1370/0/91	1313/0/91
Goodness-of-fit on F^2	1.0845	1.0660	1.1420	1.1010
R_1 [I>2σ(I)]	0.0235	0.0359	0.0334	0.0327
wR_2 [I>2σ(I)]	0.0571	0.0836	0.0936	0.0846
R_1 (all data)	0.0294	0.0449	0.0389	0.0389
wR_2 (all data)	0.0596	0.0900	0.0971	0.0876

S7. Temperature-Dependent Fits and Coupled Mode Analysis

To analyze the temperature evolution of the two A_g modes, we followed the framework by Benshalom *et al.*[17].

The Raman scattering intensity of a crystal is described by the fourth-rank tensor:

$$I(\Omega, T)_{\mu\nu\xi\rho} = \sum_{\lambda\lambda'} \chi_{\lambda}^{*\mu\nu} \chi_{\lambda'}^{\xi\rho} [n(\Omega, T) + 1] J_{\lambda\lambda'}(\Omega), \tag{S10}$$

where $n(\Omega, T)$ is the Bose-Einstein occupation factor, and $\chi_{\lambda}^{\mu\nu}$ is the susceptibility derivatives along mode λ, and the indices μ, ν are cartesian components for the induced dielectric polarization and incident field, respectively. $J_{\lambda\lambda'}(\Omega)$ is the spectral function that encodes the lineshape and will be discussed below.

Experimentally, all measurements were performed with the same incident linear polarization, and the scattered light was collected in the parallel configuration. Also, each spectrum was reduced by the Bose-Einstein factor $[n(\Omega, T) + 1]$, allowing Eq. (S10) to be simplified to:

$$I(\Omega) = \sum_{\lambda\lambda'} \chi_{\lambda} \chi_{\lambda'} J_{\lambda\lambda'}(\Omega), \tag{S11}$$

where χ_{λ} are the effective susceptibility coefficients in the measured configuration.

Spectral Function via Green's Function Formalism

To model the spectral function $J_{\lambda\lambda'}(\Omega)$, we begin with the single-particle retarted Green's function $G(\Omega)$, which characterizes the ionic motion in the crystal[18]. The spectral function is related to the imaginary part of the Green's function by:

$$J_{\lambda\lambda'}(\Omega, T) = -\frac{1}{\pi} \mathrm{Im}\{G_{\lambda\lambda'}(\Omega)\}. \tag{S12}$$

The Green's function is expressed through the harmonic Green's function $g^0(\Omega)$ and the self-energy matrix $\Sigma(\Omega)$ via the Dyson equation:

$$G^{-1}(\Omega) = \left[g^0(\Omega)\right]^{-1} + \Sigma(\Omega). \tag{S13}$$

For the case of two modes, the we model the self-energy as:

$$g^0(\Omega) = \begin{pmatrix} \frac{2\omega_{A_g,1}}{\Omega^2-\omega^2_{A_g,1}} & 0 \\ 0 & \frac{2\omega_{A_g,2}}{\Omega^2-\omega^2_{A_g,2}} \end{pmatrix}, \quad \Sigma(\Omega) = \begin{pmatrix} i\Gamma_{A_g,1} & \gamma \\ \gamma & i\Gamma_{A_g,2} \end{pmatrix}, \tag{S14}$$

where $\omega_{A_g,1}, \omega_{A_g,2}$ and $\Gamma_{A_g,1}, \Gamma_{A_g,2}$ are the vibrational frequencies and damping rates of the two modes, respectively. The off-diagonal self-energy coupling element γ introduces correlation between the modes.

Uncoupled Modes Fit: no cross-correlation between the modes

The standard approach for modeling a Raman spectrum relies on the quasi-harmonic approximation, which incorporates thermal expansion (temperature-dependent frequency shifts) and finite vibrational lifetimes (non-zero diagonal self-energy components Γ). In this approximation, modes are treated as orthogonal and uncorrelated, implying a zero off-diagonal self-energy, $\gamma = 0$. We address the more general case of cross-correlation between the modes ($\gamma \neq 0$) in a subsequent section.

Assuming viscous damping[19], the spectral function simplifies to a Lorentzian form:

$$J_\lambda(\Omega; T) = \frac{4\omega_\lambda |\Omega| \Gamma_\lambda}{(\Omega^2 - \omega_\lambda^2)^2 + 4\Gamma_\lambda^2 \Omega^2}. \tag{S15}$$

Substituting into Eq. (S11), we obtain:

$$I(\Omega) = \sum_\lambda \chi_\lambda^2 \frac{4\omega_\lambda |\Omega| \Gamma_\lambda}{(\Omega^2 - \omega_\lambda^2)^2 + 4\Gamma_\lambda^2 \Omega^2}. \tag{S16}$$

This expression was used to fit the reduced Raman spectra shown in Figures 4a–c, S8, and S9 (red curves). The resulting fitting parameters are found in Table S4.

Table S4: Fit results to the uncoupled modes model, $\gamma = 0$

Temperature [K]	ω_1	ω_2	Γ_1	Γ_2	χ_1	χ_2
80	491.7078	520.4174	1.0768	1.1686	2.2717	1.6500
90	491.7217	520.4364	1.0710	1.1569	2.2677	1.6467
100	491.6727	520.1169	1.1211	1.2520	2.2591	1.6458
110	491.5962	519.6505	1.1965	1.3871	2.2324	1.6468
120	491.5021	519.1037	1.2860	1.5585	2.2630	1.6824
130	491.3944	518.4981	1.3956	1.7794	2.2312	1.6790
140	491.2716	517.8299	1.5061	2.0283	2.1611	1.6518
150	491.1653	517.1427	1.6547	2.3123	2.2215	1.7260
160	491.0463	516.4333	1.8126	2.6179	2.1914	1.7290
170	490.9280	515.6553	2.0011	2.9737	2.1760	1.7541
180	490.8425	514.8398	2.2016	3.3786	2.1527	1.7739
190	490.7386	513.9575	2.4254	3.7733	2.1257	1.7952
200	490.6734	512.9989	2.6808	4.2369	2.1031	1.8245
210	490.6064	511.7513	3.0107	4.8270	2.0956	1.8880
220	490.6262	510.4187	3.3918	5.3938	2.0704	1.9342
230	490.6555	509.3298	3.7008	5.7589	2.0452	1.9717
240	490.7232	508.2743	3.9649	6.0509	2.0093	1.9951
250	490.8198	507.2480	4.2227	6.2998	1.9741	2.0127
260	490.9659	506.2689	4.5215	6.4456	1.9367	2.0320
270	491.1528	505.3736	4.7391	6.5048	1.9107	2.0534
280	491.3630	504.4645	4.9442	6.5712	1.8657	2.0717
290	491.5903	503.6996	5.1199	6.5507	1.8383	2.0856
300	491.7769	502.8876	5.2219	6.5229	1.7803	2.0957
310	492.1712	502.3891	5.3884	6.3534	1.7677	2.0733
320	492.3927	501.7448	5.4783	6.3121	1.7214	2.0704
330	492.6490	501.2094	5.4991	6.2293	1.6851	2.0691
340	492.8473	500.7104	5.5113	6.1405	1.6288	2.0457
350	493.1197	500.3452	5.5356	5.9913	1.6077	1.9962
360	493.4270	500.0009	5.5187	5.9320	1.5955	1.9740
370	493.8959	499.9103	5.5444	5.7638	1.6729	1.9135
380	493.8775	499.4921	5.4341	5.7514	1.6163	1.9489
390	493.9595	499.1140	5.3119	5.7601	1.5677	1.9674
400	494.3955	499.2504	5.3826	5.6208	1.7231	1.8399
410	494.4275	498.9656	5.2943	5.6559	1.7217	1.8497
420	494.2771	498.6842	5.2000	5.6347	1.6304	1.8309
430	494.7754	499.1119	5.3367	5.5975	1.7706	1.5355
440	494.6235	498.8151	5.3303	5.5883	1.7800	1.5538

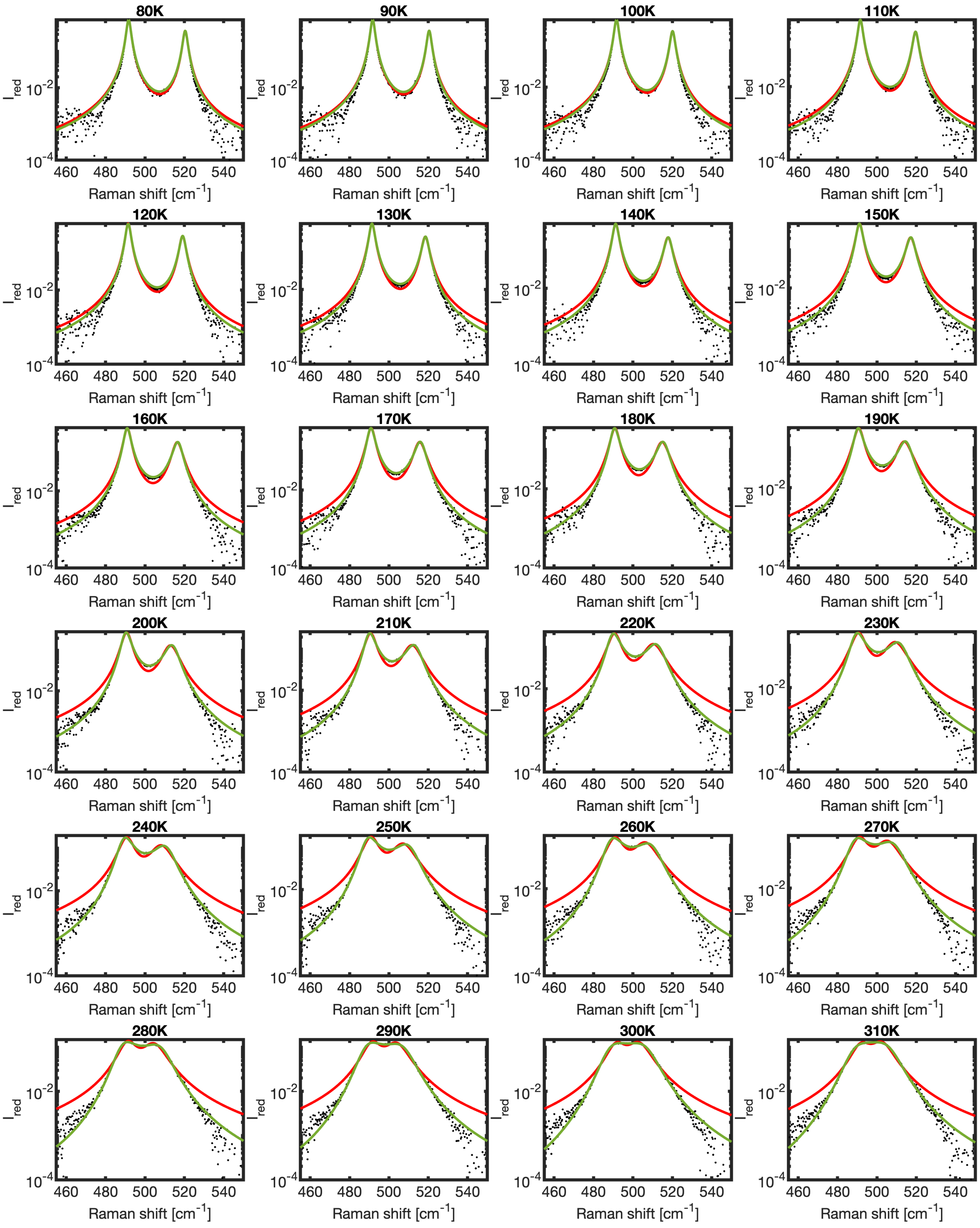
80K
90K
100K
110K
120K
130K
140K
150K
160K
170K
180K
190K
200K
210K
220K
230K
240K
250K
260K
270K
280K
290K
300K
310K
I_{red}
10^{-2}
10^{-4}
460 480 500 520 540
Raman shift [cm^{-1}]

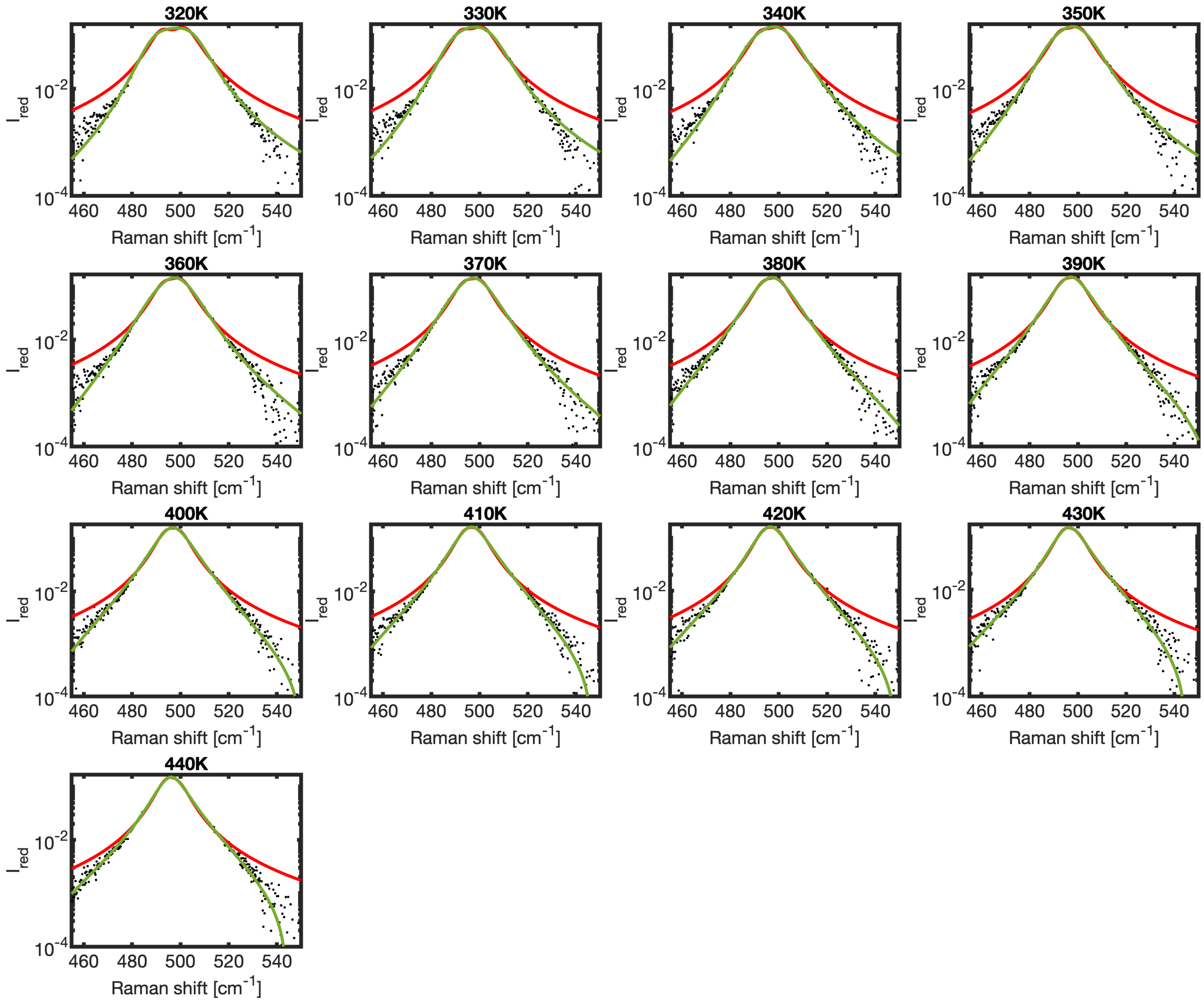

Figure S8: Temperature dependent Raman spectra (semilog scale) of ω_1 and ω_2 reduced to the Bose-Einstein occupation factor (black points), and the fits to a sum of uncoupled modes ($\gamma = 0$,red curve) and two coupled modes ($\gamma \neq 0$,green curve).

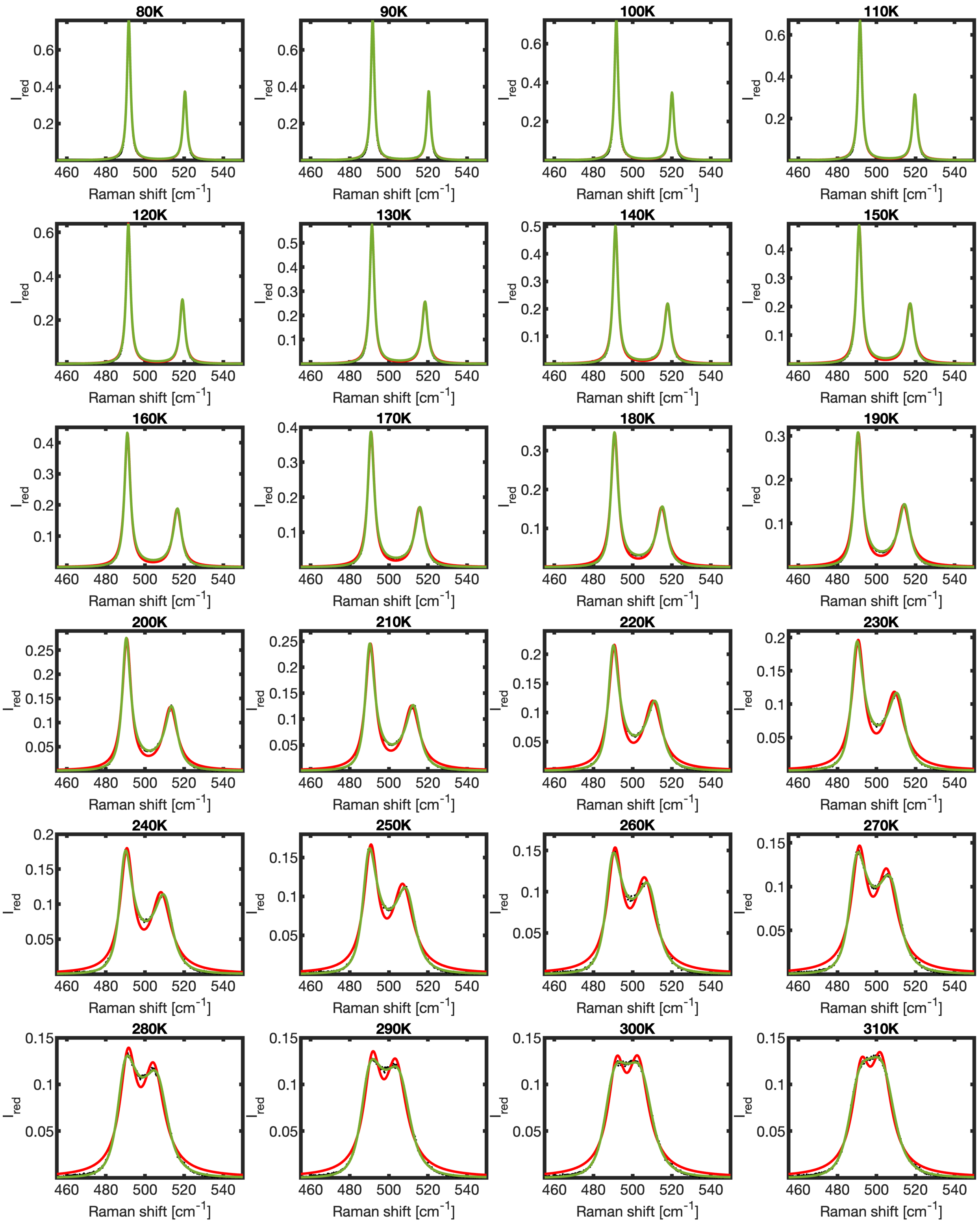
80K
90K
100K
110K
120K
130K
140K
150K
160K
170K
180K
190K
200K
210K
220K
230K
240K
250K
260K
270K
280K
290K
300K
310K
I_{red}
Raman shift [cm^{-1}]

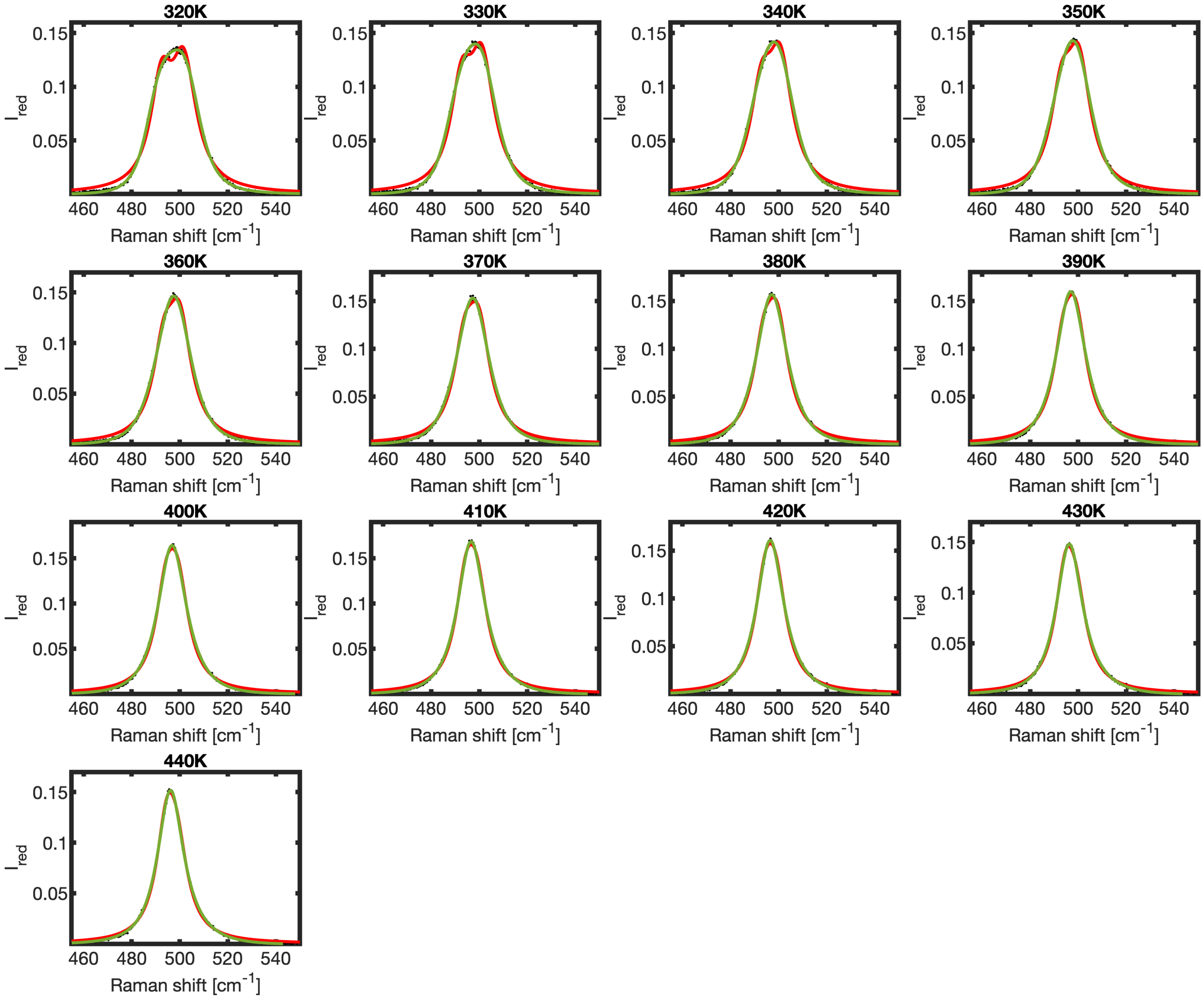

Figure S9: Temperature dependent Raman spectra (linear scale) of ω_1 and ω_2 reduced to the Bose-Einstein occupation factor (black points), and the fits to a sum of uncoupled modes ($\gamma = 0$,red curve) and two coupled modes ($\gamma \neq 0$,green curve).

Coupled Modes Fit: cross-correlation between the modes

While Eq. (S16) provides an adequate fit to the low-temperature spectra, it fails to capture the spectral features at higher temperatures, as evident in Figure 4b–c and in the high-temperature-resolution data in Figure S8, S9. Specifically, it overestimates the peak intensities and underestimates the central dip across intermediate temperatures. To address this, we allow a non-zero off-diagonal self-energy component, $\gamma \neq 0$ in Eq. (S14), introducing temperature-dependent cross-correlation between the modes. Using purely imaginary-valued γ yields satisfactory fits across the entire temperature range. The resulting coupled-mode fits are shown as green curves in Figure 4a–c and Figures S8, S9. The temperature dependence of the fit parameters (ω_1, ω_2, Γ_1, Γ_2, χ_1, χ_2, γ) is presented in Fig. S10, and Table S5.

Table S5: Fit results to the coupled modes model, $\gamma \neq 0$

Temperature [K]	ω_1	ω_2	Γ_1	Γ_2	χ_1	χ_2	γ
80	491.6934	520.4454	1.0755	1.1641	2.2720	1.6485	-0.2214i
90	491.7093	520.4607	1.0700	1.1529	2.2681	1.6454	-0.1953i
100	491.6576	520.1470	1.1197	1.2471	2.2593	1.6442	-0.2230i
110	491.5663	519.7087	1.1934	1.3776	2.2323	1.6436	-0.3792i
120	491.4589	519.1892	1.2813	1.5428	2.2622	1.6773	-0.4870i
130	491.3306	518.6260	1.3881	1.7536	2.2292	1.6709	-0.6262i
140	491.1817	518.0120	1.4953	1.9879	2.1574	1.6402	-0.7741i
150	491.0343	517.4068	1.6380	2.2494	2.2149	1.7085	-0.9669i
160	490.8607	516.8061	1.7880	2.5241	2.1809	1.7046	-1.1852i
170	490.6727	516.1635	1.9678	2.8410	2.1608	1.7216	-1.4071i
180	490.4896	515.5376	2.1577	3.1858	2.1316	1.7299	-1.6786i
190	490.2702	514.8600	2.3711	3.5282	2.0982	1.7412	-1.9310i
200	490.0374	514.1978	2.6201	3.9113	2.0685	1.7555	-2.2669i
210	489.6929	513.4118	2.9512	4.4321	2.0503	1.8012	-2.7366i
220	489.3511	512.6314	3.3663	4.9736	2.0171	1.8307	-3.2331i
230	489.0319	512.0001	3.7334	5.3934	1.9866	1.8586	-3.6435i
240	488.7040	511.4231	4.0918	5.7964	1.9467	1.8749	-4.0777i
250	488.3346	510.9282	4.4924	6.2330	1.9085	1.8876	-4.5696i
260	487.9210	510.4386	5.0043	6.6489	1.8704	1.9048	-5.0855i
270	487.5768	509.9532	5.4596	7.0234	1.8418	1.9278	-5.5454i
280	487.0513	509.6565	6.0632	7.4731	1.8028	1.9391	-6.2476i
290	486.5928	509.3265	6.6443	7.8943	1.7743	1.9569	-6.8410i
300	486.0291	509.0355	7.3396	8.3419	1.7319	1.9588	-7.5394i
310	485.7568	508.8080	7.9942	8.6985	1.7112	1.9512	-8.0730i
320	485.0961	508.7908	8.8829	9.2915	1.6811	1.9403	-8.9832i
330	484.6214	508.7128	9.7204	9.8671	1.6574	1.9376	-9.7530i
340	483.8450	509.0682	10.7649	10.6216	1.6279	1.8980	-10.9528i
350	483.4238	508.9089	11.5036	11.0997	1.5868	1.8742	-11.5961i
360	482.5929	510.3797	12.9152	12.6641	1.6240	1.8113	-13.6519i
370	482.5370	510.8999	13.5804	13.5603	1.6596	1.7883	-14.5457i
380	481.8191	512.6598	14.6966	15.3903	1.7033	1.7342	-16.6057i
390	480.7176	515.0256	16.0930	17.8836	1.7391	1.6772	-19.3222i
400	480.5210	516.7770	16.4271	19.5128	1.7954	1.6249	-20.7390i
410	480.0667	519.8546	16.9585	22.2884	1.8657	1.5525	-23.0621i
420	480.2208	519.5955	17.0917	22.7203	1.8132	1.5079	-23.1028i
430	479.7983	523.5597	17.1810	25.5903	1.7986	1.3551	-25.3957i
440	478.7182	525.9741	17.9705	27.8700	1.8349	1.3374	-27.5776i

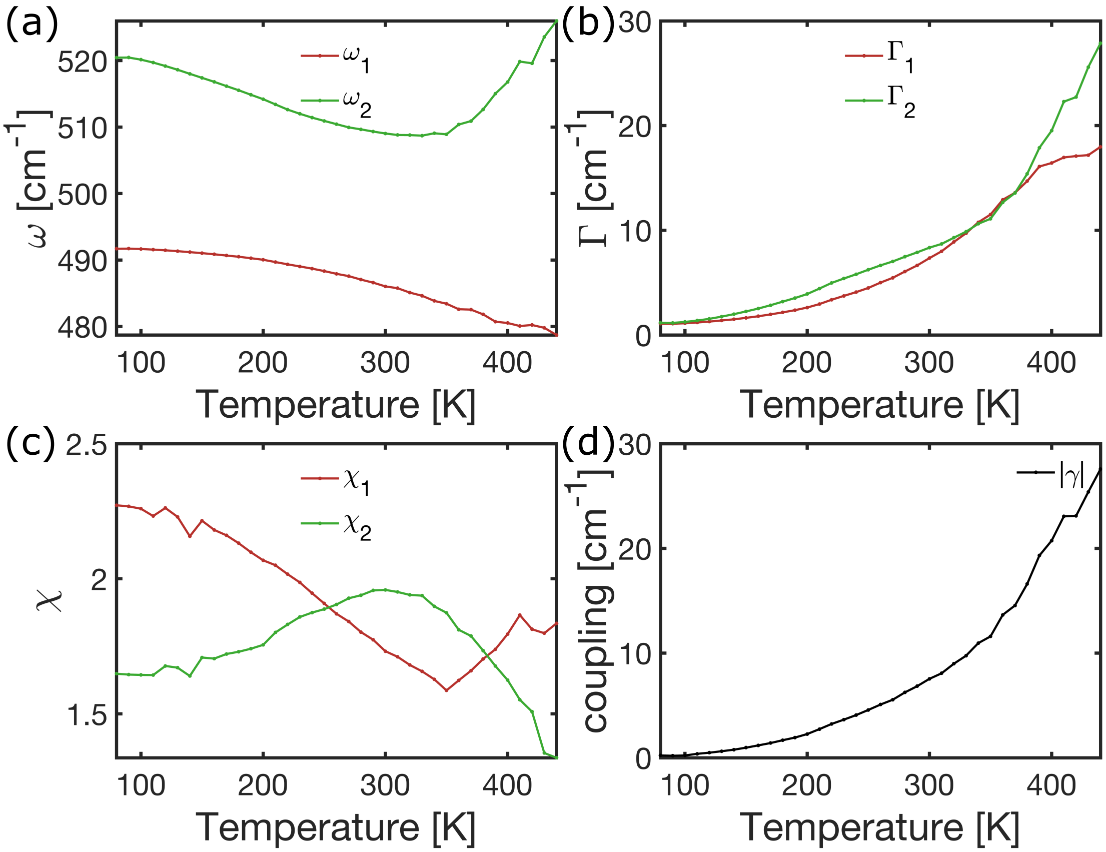

Figure S10: Coupled modes model parameter evolution as a function of temperature.

Pseudo-Voigt fit

To verify that the observed asymmetric lineshape does not originate from the system response, we fit the Raman spectra at selected temperatures using a sum of two Pseudo-Voigt profiles, as described by Eq. (S17):

$$I(\Omega) = \sum_{\lambda} \chi_{\lambda}^{2} \left[(1-n_{\lambda}) \cdot \frac{4\omega_{\lambda}|\Omega|\Gamma_{\lambda}}{(\Omega^{2}-\omega_{\lambda}^{2})^{2}+4\Gamma_{\lambda}^{2}\Omega^{2}} + n_{\lambda} \cdot \exp\left(-\frac{(|\Omega|-\omega_{\lambda})^{2}\ln 16}{(2\Gamma_{\lambda})^{2}} \right) \right]. \qquad \text{(S17)}$$

where the additional parameters n_λ varies from 0–1 and quantifies the ratio between the Lorentzian and Gaussian contributions.

Figure S11 compares the fit at three temperatures, of two pseudo-Voigt peaks with the uncoupled modes model ($\gamma = 0$, Eq. (S16)), showing an improved fit using two pseudo-Voigt peaks, supporting that two normal modes is inadequate to describe the spectral lineshape. Next, we compare the pseudo-Voigt accounting for system response, with the coupled modes model ($\gamma \neq 0$) which accounts for cross-correlation between the two modes. The pseudo-Voigt model, shown in Eq. (S17), involves eight fitting parameters in total,

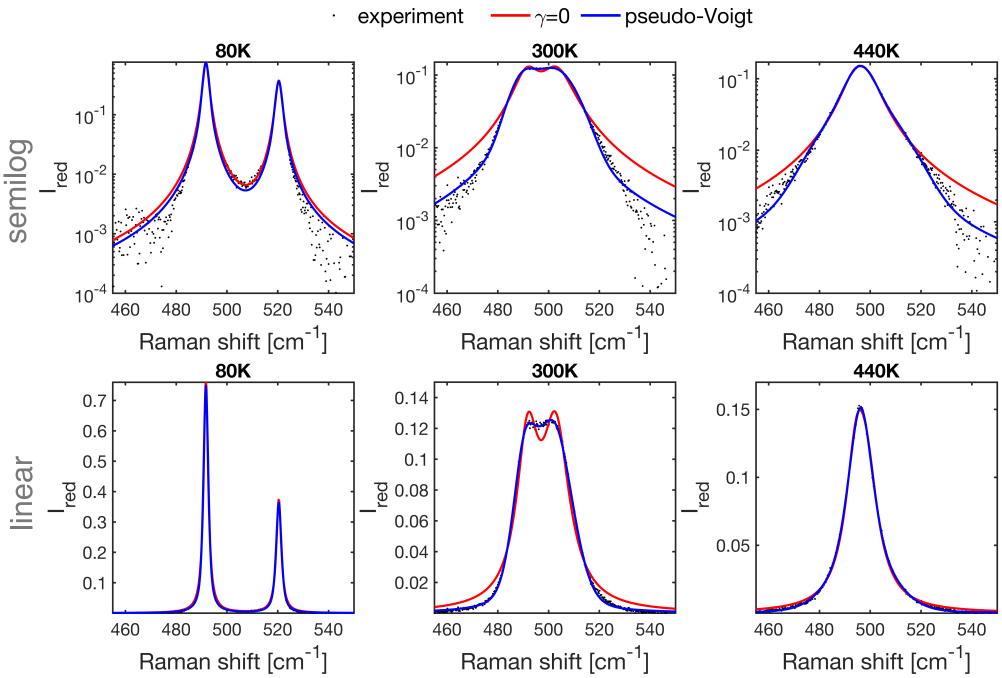

Figure S11: Comparison between the fits to the experimental data (black dots) of two uncoupled modes ($\gamma = 0$, red curve) with two pseudo-Voigt peaks (blue curve). The top row is the plots in semilog scale and the bottom plots are in linear scale.

whereas the coupled modes model with $\gamma \neq 0$ requires only seven. The results of both fits for the full temperature range is shown in Figure S12, S13 on semilogarithmic and linear scales, respectively. While the coupled-modes model fits the data well at all temperatures, the fit to two pseudo-Voigt peaks yields comparable fits at low and high temperatures but misses in most intermediate temperatures.

Given that the coupled modes model not only provides better overall fit quality but also requires fewer parameters, we conclude that it is the more appropriate description. This supports the interpretation that the asymmetric lineshape arises from physical mode coupling rather than the instrumental response.

Table S6: Fit results to the two pseudo-Voigt peaks

Temperature [K]	ω_1	ω_2	Γ_1	Γ_2	χ_1	χ_2	n_1	n_2
80	491.7050	520.4129	1.1703	1.2646	2.2310	1.6080	0.2767	0.2301
90	491.7183	520.4318	1.1637	1.2515	2.2272	1.6052	0.2751	0.2319
100	491.6701	520.1137	1.2126	1.3418	2.2250	1.6085	0.2501	0.1865
110	491.5952	519.6478	1.2812	1.4669	2.2056	1.6174	0.2050	0.1330
120	491.5014	519.1005	1.3635	1.6297	2.2414	1.6599	0.1611	0.0921
130	491.3944	518.4943	1.4661	1.8494	2.2141	1.6609	0.1250	0.0687
140	491.2718	517.8263	1.5658	2.0826	2.1464	1.6424	0.0910	0.0383
150	491.1662	517.1381	1.7139	2.3686	2.2060	1.7199	0.0752	0.0290
160	491.0478	516.4285	1.8609	2.6643	2.1787	1.7269	0.0510	0.0175
170	490.9293	515.6508	2.0394	3.0132	2.1664	1.7538	0.0327	0.0111
180	490.8439	514.8342	2.2299	3.4182	2.1483	1.7718	0.0195	0.0090
190	490.7405	513.9498	2.4576	3.8194	2.1200	1.7948	0.0186	0.0078
200	490.6763	512.9840	2.7111	4.3311	2.1112	1.8105	0.0121	0.0155
210	490.6217	511.6807	3.0839	5.2434	2.1797	1.7953	0.0147	0.0638
220	490.6678	510.0115	3.7160	6.9784	2.7249	1.5215	0.0771	0.4379
230	490.6462	508.8261	4.1756	7.5486	3.0871	1.4589	0.1419	0.8313
240	490.6162	507.8075	4.4656	7.7879	3.0385	1.4728	0.1768	0.9223
250	490.5865	506.8630	4.7190	7.9438	2.7580	1.5259	0.2007	0.7824
260	490.5735	505.9733	4.9980	7.9835	2.3872	1.6275	0.2135	0.5512
270	490.5986	505.1268	5.1964	8.0087	2.1356	1.7395	0.2258	0.4076
280	490.5808	504.2051	5.3926	8.1373	2.0048	1.8016	0.2188	0.3586
290	490.5743	503.3552	5.5752	8.2337	1.8580	1.8925	0.2226	0.3029
300	490.4701	502.3572	5.7277	8.4082	1.7329	1.9454	0.2091	0.2811
310	490.7182	501.8190	6.0556	8.2710	1.5783	2.0516	0.2389	0.2326
320	490.6239	500.8292	6.3478	8.4925	1.4110	2.1394	0.2497	0.2196
330	493.1450	501.7872	7.9261	8.1955	1.3428	2.7800	0.7608	0.1135
340	494.5116	500.4570	8.6908	8.8745	1.2851	3.0050	0.9387	0.1046
350	494.1866	499.3832	8.4200	8.5587	1.0994	3.2014	0.9497	0.1029
360	493.4801	498.3008	8.3771	8.2973	0.8046	3.4691	0.9645	0.1158
370	497.4029	497.2602	13.4485	6.8139	1.2079	3.2151	0.9997	0.2622
380	497.4455	497.1170	13.4451	6.4302	1.2273	3.1966	0.9990	0.2643
390	497.4372	496.9594	13.0390	6.0541	1.2174	3.3086	0.9999	0.2132
400	497.3486	496.8218	13.1122	5.8950	1.2175	3.3277	0.9994	0.2215
410	497.3634	496.6612	13.0943	5.7416	1.2107	3.3903	0.9993	0.2147
420	497.3164	496.5097	13.2292	5.6654	1.1518	3.3934	0.9992	0.2025
430	497.3221	496.3676	13.1481	5.5841	1.0950	3.2968	1.0000	0.1869
440	497.0658	496.1965	13.1448	5.5420	1.0747	3.4661	1.0000	0.1602

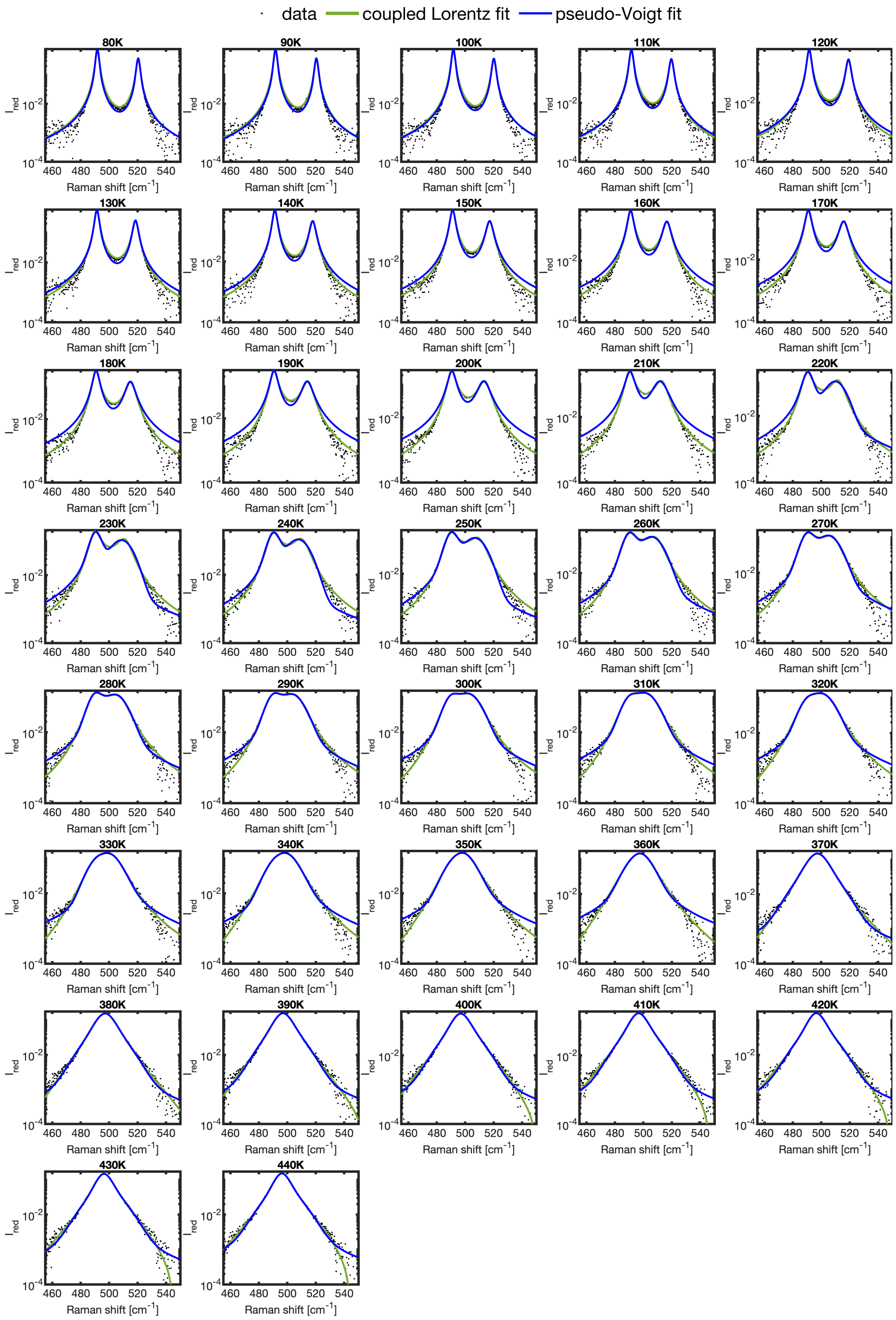

Figure S12: Comparison between the fits to two pseudo-Voigt peaks (blue) and two coupled modes (green) in semilog scale.

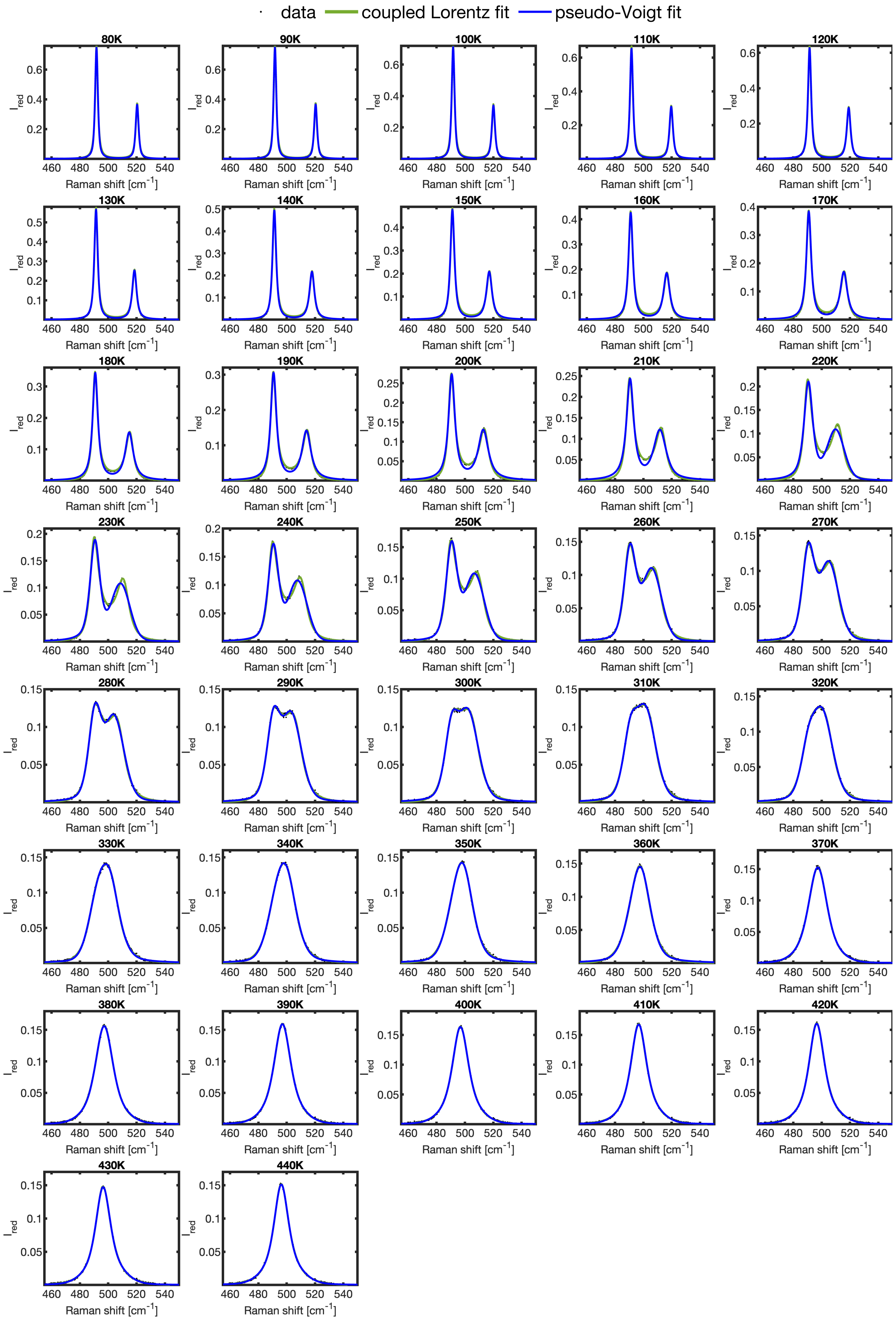

Figure S13: Comparison between the fits to two pseudo-Voigt peaks (blue) and two coupled modes (green) in linear scale.

S8. Numerical Simulation of the Spectral Function

To simulate the spectral function, we constructed a one-dimensional double-well potential using the same parameters as the `eig_double_well.m` MATLAB script[20], available on the Book Site[21]. This script employs an adiabatic switching approach: it begins with a harmonic potential for which the eigenstates and eigenenergies are analytically known and incrementally deforms the potential toward a double-well geometry, recalculating the eigenstates and eigenenergies at each step. We introduced several modifications to the original code: (1) added an asymmetric component to the potential, (2) reduced the deformation step size and increased the number of steps, (3) extended the number of computed eigenstates and eigenenergies to 27, and (4) added a validation routine to ensure normalization of the eigenfunctions.

Figure S14a shows the initial harmonic potential $V_{har}(x) = 4.59\,x^2$ (gray), and the modification for a symmetric double-well potential $V_{\text{sym}}(x) = e^{-18.36x^2}(-30\,x^2+1)$ (light blue). To introduce asymmetry, we added a linear term $V_{\text{asym}}(x) = -0.0464\,x - 0.0138$ on the portion of the potential between the maximum and minimum on the negative x-axis (dashed orange). The final potential is a sum of the harmonic potential and its modifications:

$$V(x) = V_{har(x)} + V_{sym}(x) + a \cdot V_{asym}(x) \tag{S18}$$

where the strength of the asymmetry is controlled by a parameter a, ranging from $a = 0$, fully symmetric to $a = 1$, maximal asymmetry, as illustrated in Fig. S14b. Notably, the depth of the wells and the height of the barrier stay constant throughout all of the simulation.

The simulated spectrum is computed as a sum over transitions between eigenstates:

$$I(\Omega) = \sum_{j>i} n_i \cdot \left|\langle \psi_j \mid x \mid \psi_i \rangle\right|^2 \cdot \mathcal{G}(\Omega, (E_j - E_i), 0.001) \tag{S19}$$

where $n_i = \exp\left(-\frac{E_i}{E_1} \cdot R\right)$ is the Boltzmann population of state i, choosing $R = \frac{E_1}{kT} = 10$ so that only transitions that are below the barrier are accounted for, and $\left|\langle \psi_j \mid x \mid \psi_i \rangle\right|^2$ is the

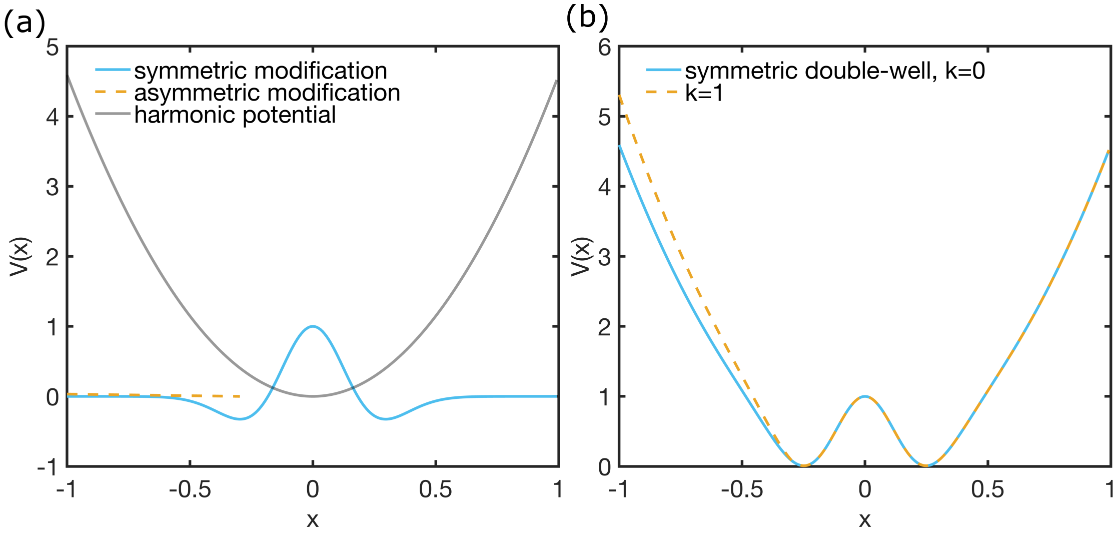

Figure S14: (a) Potential energy curves used in the simulation. Gray: initial harmonic potential. Light blue: symmetric double-well. Dashed orange: asymmetric modification. (b) Interpolation between symmetric ($a = 0$) and asymmetric ($a = 1$) potentials.

squared dipole matrix element. The function $\mathscr{G}$ is a normalized Gaussian:

$$\mathscr{G}(\Omega, \omega, \sigma) = \frac{1}{2\pi\sigma} \exp\left(-\frac{(\Omega - \omega)^2}{2\sigma^2}\right) \tag{S20}$$

with $\omega = E_j - E_i$ and the choice of $\sigma = 0.001$ to achieve adequate spectral resolution.

Figure S15 displays the eigenenergies (red dashed lines) for a symmetric double-well ($a = 0$, left panel) and the most asymmetric case considered ($a = 1$, right panel). To focus on the most probable transitions that are in close proximity in frequency, we applied the following filtering steps: (1) transitions from states with negligible thermal population were discarded, (2) transitions with near-degenerate energy levels were excluded, and (3) transitions with matrix elements below a numerical threshold were removed. The black arrows indicate the transitions with a significant contribution to the spectral function. This procedure was repeated for twenty-one potentials with a varying from 0 to 1 in equally distant increments of 0.5. The resulting spectra at $a = 0.4$ to $a = 1$ in steps of 0.1 are shown in Figure 5 of the main text, revealing the gradual merging of the two peaks as the potential becomes more symmetric and the transition energies converge.

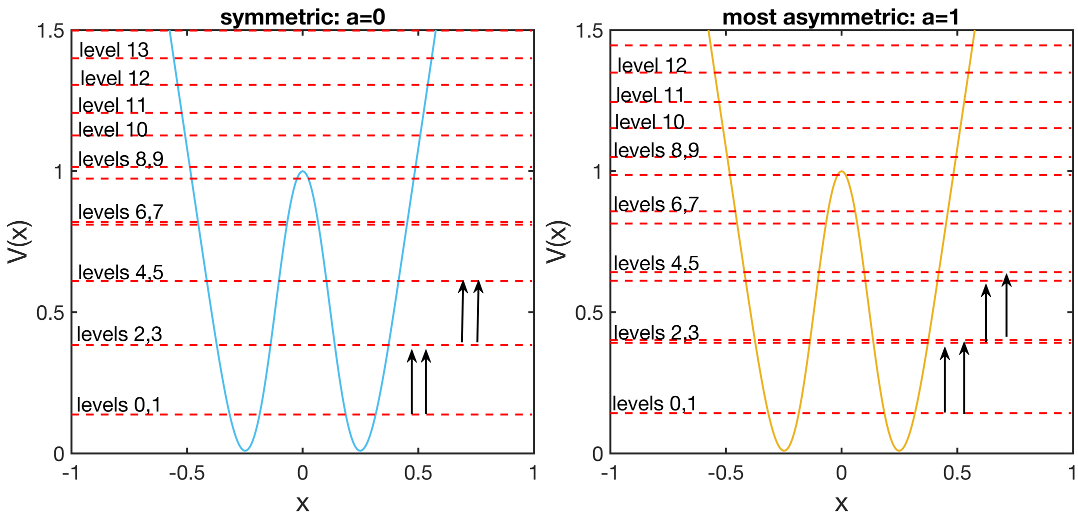

Figure S15: Eigenenergies (red dashed lines) of a symmetric double-well potential (left) and an asymmetric double-well potential (right). Black arrows indicate transitions with significant contributions to the spectrum. In the symmetric case, transitions occur between degenerate pairs of levels, resulting in closely spaced transition energies. In the asymmetric case, the energy splitting between adjacent levels lifts the degeneracy, leading to distinct transition energies.

References

[1] E. Meirzadeh, L. Sapir, H. Cohen, S. R. Cohen, D. Ehre, D. Harries, M. Lahav, and I. Lubomirsky, Nonclassical Crystal Growth as Explanation for the Riddle of Polarity in Centrosymmetric Glycine Crystals, Journal of the American Chemical Society **138**, 14756 (2016).

[2] M. Asher, D. Angerer, R. Korobko, Y. Diskin-Posner, D. A. Egger, and O. Yaffe, Anharmonic Lattice Vibrations in Small-Molecule Organic Semiconductors, Advanced Materials **32**, 1908028 (2020).

[3] G. Kresse and J. Furthmüller, Efficient Iterative Schemes for *Ab Initio* Total-Energy Calculations Using a Plane-Wave Basis Set, Physical Review B **54**, 11169 (1996).

[4] J. P. Perdew, K. Burke, and M. Ernzerhof, Generalized Gradient Approximation Made Simple, Physical Review Letters **77**, 3865 (1996).

[5] S. Grimme, J. Antony, S. Ehrlich, and H. Krieg, A Consistent and Accurate Ab Initio Parametrization of Density Functional Dispersion Correction (DFT-D) for the 94 Elements H–Pu, The Journal of Chemical Physics **132**, 154104 (2010).

[6] S. Grimme, S. Ehrlich, and L. Goerigk, Effect of the Damping Function in Dispersion Corrected Density Functional Theory, Journal of Computational Chemistry **32**, 1456 (2011).

[7] E. V. Boldyreva, T. N. Drebushchak, and E. S. Shutova, Structural Distortion of the α, β, and γ Polymorphs of Glycine on Cooling, Zeitschrift für Kristallographie - Crystalline Materials **218**, 366 (2003).

[8] A. Togo, L. Chaput, T. Tadano, and I. Tanaka, Implementation Strategies in Phonopy and Phono3py, Journal of Physics: Condensed Matter **35**, 353001 (2023).

[9] A. Togo, First-Principles Phonon Calculations with Phonopy and Phono3py, Journal of the Physical Society of Japan **92**, 012001 (2023).

[10] P. Y. Yu and M. Cardona, *Fundamentals of Semiconductors: Physics and Materials Properties* (Springer Science & Business Media, 2010) pp. 375–385.

[11] C. Kranert, C. Sturm, R. Schmidt-Grund, and M. Grundmann, Raman Tensor Formalism for Optically Anisotropic Crystals, Physical Review Letters **116**, 127401 (2016).

[12] D. L. Rousseau, R. P. Bauman, and S. P. S. Porto, Normal Mode Determination in Crystals, Journal of Raman Spectroscopy **10**, 253 (1981).

[13] G. M. Sheldrick, Crystal Structure Refinement with *SHELXL*, Acta Crystallographica Section C **71**, 3 (2015).

[14] G. M. Sheldrick, *SHELXT* – Integrated Space-Group and Crystal-Structure Determination, Acta Crystallographica Section A **71**, 3 (2015).

[15] O. V. Dolomanov, L. J. Bourhis, R. J. Gildea, J. A. K. Howard, and H. Puschmann, OLEX2: A Complete Structure Solution, Refinement and Analysis Program, Journal of Applied Crystallography **42**, 339 (2009).

[16] F. Kleemiss, O. V. Dolomanov, M. Bodensteiner, N. Peyerimhoff, L. Midgley, L. J. Bourhis, A. Genoni, L. A. Malaspina, D. Jayatilaka, J. L. Spencer, *et al.*, Accurate Crystal Structures and Chemical Properties from NoSpherA2, Chemical Science **12**, 1675 (2021).

[17] N. Benshalom, M. Asher, R. Jouclas, R. Korobko, G. Schweicher, J. Liu, Y. Geerts, O. Hellman, and O. Yaffe, Phonon–Phonon Interactions in the Polarization Dependence of Raman Scattering, The Journal of Physical Chemistry C **127**, 18099 (2023).

[18] P. C. K. Kwok, Green's Function Method in Lattice Dynamics, in *Solid State Physics*, Vol. 20 (Elsevier, 1968) pp. 213–303.

[19] R. K. Wehner and E. F. Steigmeier, Coupled Lattice Modes in Light Scattering, RCA Review **36**, 70 (1975).

[20] D. Tannor, *Introduction to Quantum Mechanics: A Time-Dependent Perspective* (MIT Press, 2007) pp. 199–201.

[21] D. Tannor, Matlab tdqm source codes, `https://www.weizmann.ac.il/chembiophys/tannor/matlab-tdqm-source-codes` (n.d.), accessed: 2025-07-19.
